# Identification of SFBB-Containing Canonical and Noncanonical SCF Complexes in Pollen of Apple (*Malus* × *domestica*)

**DOI:** 10.1371/journal.pone.0097642

**Published:** 2014-05-21

**Authors:** Mai F. Minamikawa, Ruriko Koyano, Shinji Kikuchi, Takato Koba, Hidenori Sassa

**Affiliations:** Graduate School of Horticulture, Chiba University, Matsudo, Chiba, Japan; Leibniz Institute of Plant Biochemistry, Germany

## Abstract

Gametophytic self-incompatibility (GSI) of Rosaceae, Solanaceae and Plantaginaceae is controlled by a single polymorphic *S* locus. The *S* locus contains at least two genes, *S-RNase* and F-box protein encoding gene *SLF/SFB/SFBB* that control pistil and pollen specificity, respectively. Generally, the F-box protein forms an E3 ligase complex, SCF complex with Skp1, Cullin1 (CUL1) and Rbx1, however, in *Petunia inflata*, SBP1 (S-RNase binding protein1) was reported to play the role of Skp1 and Rbx1, and form an SCF^SLF^-like complex for ubiquitination of non-self S-RNases. On the other hand, in *Petunia hybrida* and *Petunia inflata* of Solanaceae, *Prunus avium* and *Pyrus bretschneideri* of Rosaceae, SSK1 (SLF-interacting Skp1-like protein1) is considered to form the SCF^SLF/SFB^ complex. Here, we isolated pollen-expressed apple homologs of *SSK1* and *CUL1*, and named *MdSSK1, MdCUL1A* and *MdCUL1B*. *MdSSK1* was preferentially expressed in pollen, but weakly in other organs analyzed, while, *MdCUL1A* and *MdCUL1B* were almost equally expressed in all the organs analyzed. *MdSSK1* transcript abundance was significantly (>100 times) higher than that of *MdSBP1*. *In vitro* binding assays showed that MdSSK1 and MdSBP1 interacted with MdSFBB1-*S* ^9^ and MdCUL1, and MdSFBB1-*S* ^9^ interacted more strongly with MdSSK1 than with MdSBP1. The results suggest that both MdSSK1-containing SCF^SFBB1^ and MdSBP1-containing SCF^SFBB1^-like complexes function in pollen of apple, and the former plays a major role.

## Introduction

Self-incompatibility (SI) is a widespread genetic mechanism to prevent self-fertilization and promote outcrossing in angiosperms. In Rosaceae, Solanaceae and Plantaginaceae, gametophytic self-incompatibility (GSI) is controlled by a single *S* locus with multiple haplotypes. If the haploid pollen tube has an *S* haplotype in common with one of the two *S* haplotypes of the diploid pistil, the pollen tube is recognized as self and rejected [1]. The *S* haplotype contains at least two genes, *S-RNase* and F-box protein encoding gene *SLF/SFB/SFBB* that control pistil and pollen specificity, respectively [2]–[9]. S-RNase is considered to play a role in rejecting self-pollen by acting as a cytotoxin [10]–[12]. The pollen-part recognition factor F-box gene has been considered to be a single-copy gene in each *S* haplotype for a long time, however, in the tribe Pyreae of Rosaceae, *i.e.*, apple (*Malus* × *domestica)* and Japanese pear (*Pyrus pyrifolia),* two or three F-box genes, *SFBBs* (*S locus F-box brothers*), were implicated in pollen-part specificity because they show *S* haplotype-specific polymorphisms, linkage to the *S* locus and pollen-specific expression [13]. Subsequently, many more *SFBBs* located at the *S* locus region were identified in apple and pears [14]–[17]. In *Petunia* of Solanaceae, multiple F-box genes called *SLF*s are also involved in self/non-self discrimination of SI. Each type of SLF within an *S* haplotype interacted with a subset of non-self S-RNases to ubiquitinate them for degradation. Based on these findings, a ‘collaborative non-self recognition system by multiple factors’ model was proposed [18]. GSI of Japanese pear (*Pyrus pyrifolia*) is also considered to fit with the system like *Petunia* [16], [19]. On the other hand, in *Prunus* of Rosaceae, a single F-box gene called *SFB* was reported as a pollen *S* candidate, and a ‘self recognition model’ by a single factor was proposed [6], [16], [20], [21].

The F-box protein was first characterized as a component of an E3 ubiquitin ligase complex, SCF complex, in which the F-box protein binds substrates for ubiquitin-mediated proteolysis [22]. The canonical SCF complex comprises Skp1, Cullin1, F-box protein and Rbx1 [23]. In *Antirrhinum hispanicum* of Plantaginaceae, SLF-interacting Skp1-like1 (AhSSK1) was reported to form a canonical SCF complex with SLF and CUL1 [24]. Subsequently, in *Petunia hybrida* and *Petunia inflata* of Solanaceae, and *Prunus avium* and *Pyrus bretschneideri* of Rosaceae, SSK1 homologs were identified as components of the SCF complexes considered to be involved in GSI [25]–[28]. In *Petunia inflata,* another SLF-containing E3 ubiquitin ligase is reported to be a noncanonical SCF-like complex that includes S-RNase binding protein1 (SBP1) in place of Skp1 and Rbx1 [29], [30]. SLF has been predicted to interact with non-self S-RNases to ubiquitinate them for degradation by the 26S proteasome [29], [30]. Recently, a pollen-expressed SBP1 homolog of apple (MdSBP1) was identified for the first time in Rosaceae [31]. MdSBP1 includes a RING-HC domain and interacts with S-RNase, as for SBP1 homologs in Solanaceae; however, it still remains unclear whether MdSBP1 is a component of an SCF-like complex involved in GSI. In Rosaceae, both two putative E3 ligase complexes, *i.e.*, SBP1-containing SCF-like complex and SSK1-containing SCF, exist in pollen within a species have not been reported. The functions of SBP1 and SSK1 are an intriguing issue to be addressed in order to understand the biochemical mechanism of self/non-self S-RNase recognition by E3 ligase complexes.

As the first step toward understanding the molecular mechanism of the S-RNase-based GSI of Rosaceae, we aimed to identify putative members of the GSI-related E3 (-like) complex(es) of apple. Here, we isolated apple homologs of *SSK1* and *CUL1*s from pollen RNA by RT-PCR, and named them *MdSSK1*, *MdCUL1A* and *MdCUL1B*. Then, we examined the binding of MdSSK1 and MdSBP1 with MdCUL1s and MdSFBB1*-S* ^9^, a candidate for pollen *S* [14], [16]. *In vitro* binding assays showed that both MdSSK1 and MdSBP1 interacted with MdSFBB1*-S* ^9^ and MdCUL1, suggesting that both MdSSK1 and MdSBP1 would form SCF^SFBB^ (-like) complexes with MdSFBB and MdCUL1 in pollen of apple. We discuss the putative functions of the two types of SCF^SFBB^ (-like) complexes in S-RNase-based GSI of apple.

## Results

### Isolation of Pollen-expressed *SSK1* and *CUL1* Homologs from Apple

**Figure 1:**
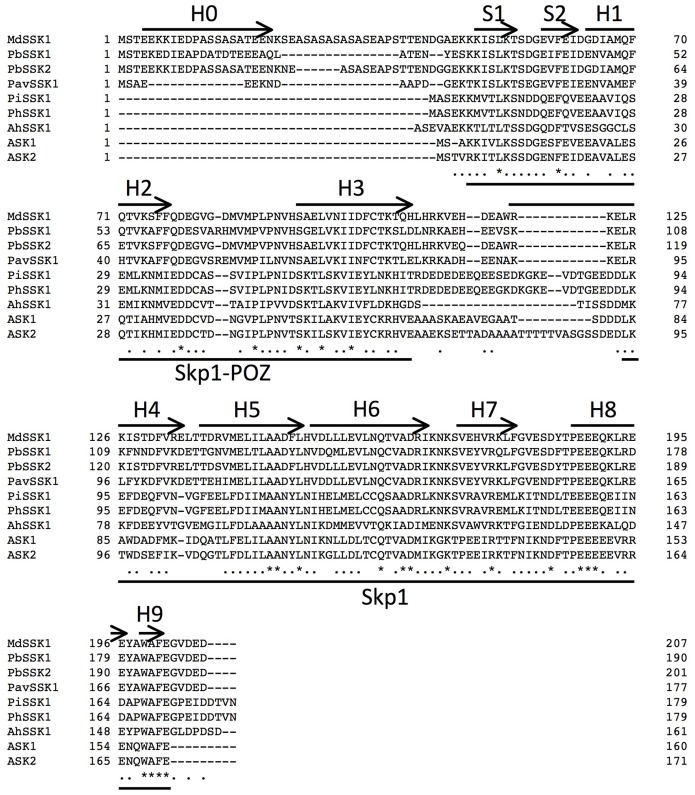
Amino acid sequence alignment of MdSSK1 and plant Skp1-like proteins. Amino acid sequences were aligned using Clustal W. MdSSK1 (AB898683), PbSSK1 (CCH26218), PbSSK2 (CCH26217), PavSSK1 (AFJ21661), PiSSK1 (AEE39461), PhSSK1 (ACT35733), AhSSK1 (ABC84199), ASK1 (NP565123), ASK2 (NP_568603) were from *Malus* × *domestica, Pyrus bretschneideri, Prunus avium*, *Petunia inflata*, *Petunia hybrida*, *Antirrhinum hispanicum* and *Arabidopsis thaliana*, respectively. Conserved sites and relatively conservative sites are marked with asterisks and dots, respectively. Arrows represent the secondary structure. S: β-sheet; H: α-helix, predicted by Phyre2 (http://www.sbg.bio.ic.ac.uk/phyre2). The Skp1-POZ and Skp1 domains detected by Pfam (http://pfam.sanger.ac.uk) are denoted by lines.

**Figure 2:**
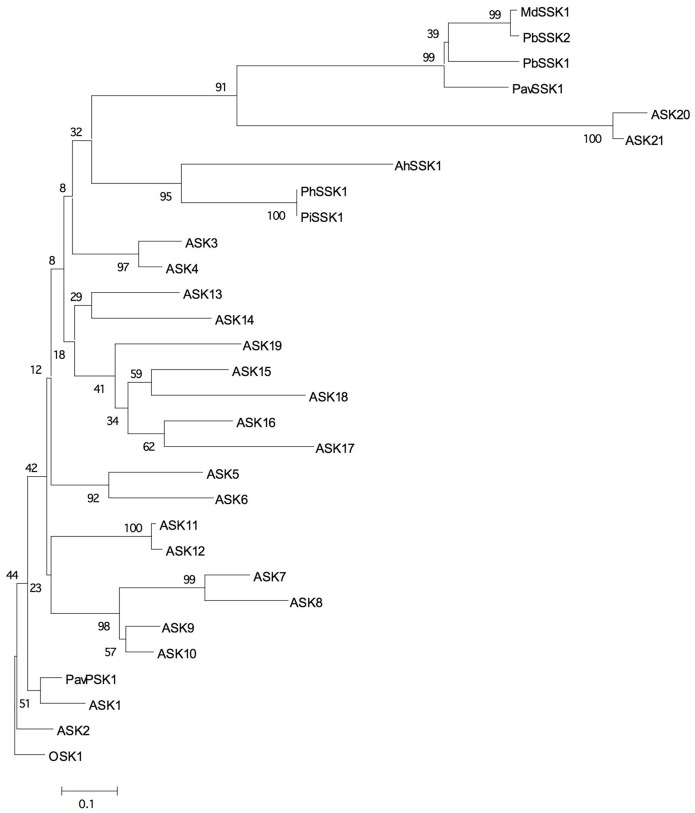
Neighbor-joining tree of MdSSK1 and other plant Skp1-like proteins. The tree was constructed based on the aligned deduced amino acid sequences from apple (MdSSK1), *Pyrus bretschneideri* (PbSSK1, PbSSK2), *Prunus avium* (PavSSK1, PavPSK1), *Petunia* (PiSSK1, PhSSK1), *Antirrhinum* (AhSSK1) and *Arabidopsis* (ASK1-21). The tree was generated with 1000 bootstrap replicates. OSK1 (LOC_Os11g26910) was defined as the outgroup.

We obtained an apple *SSK1* homolog by RT-PCR using apple pollen and named it *MdSSK1* (Figure 1, S1). The amino acid identities among SSK1 proteins were 30.6–98.8% (Table 1). Phylogenetic analysis revealed that MdSSK1 fell into a monophyletic clade of SSK1 homologs of Rosaceae (Figure 2, S1). Sequence analysis showed that MdSSK1 included two probable protein-protein interaction domains, Skp1-POZ and Skp1 domains (Figure 1), the same as solanaceous and rosaceous SSK1 proteins [24]–[27]. The Skp1-POZ and Skp1 domains were reported to interact with CUL1 and the F-box domain, respectively [32], [33].

**Table 1:** Amino acid identities (%) among MdSSK1 and other plant Skp1-like proteins.

	PbSSK1	PbSSK2	PavSSK1	PiSSK1	PhSSK1	AhSSK1	ASK1
MdSSK1	68.1	93.2	69.3	31.2	30.6	33.7	38.9
PbSSK1	–	67.1	76.0	32.0	32.0	38.0	41.6
PbSSK2		–	70.4	31.8	31.2	34.3	38.3
PavSSK1			–	31.2	32.3	35.3	40.2
PiSSK1				–	98.8	45.4	41.8
PhSSK1					–	45.4	41.8
AhSSK1						–	40.5

We also obtained two *CUL1* homologs by RT-PCR using apple pollen RNA and named them *MdCUL1A* and *MdCUL1B* (Figure S2 and S3). The two MdCUL1s showed 68.5% amino acid identity (Table S1). The amino acid identities among CUL1 proteins were 58.4∼99.4% (Table S1). Sequence analysis showed that MdCUL1A and MdCUL1B included a NEDD8 domain implicated in E3 ligase activity [34], [35], like solanaceous and rosaceous CUL1 proteins (Figure S3).

### Expression Patterns of *MdSSK1* and *MdCUL1s*

**Figure 3:**
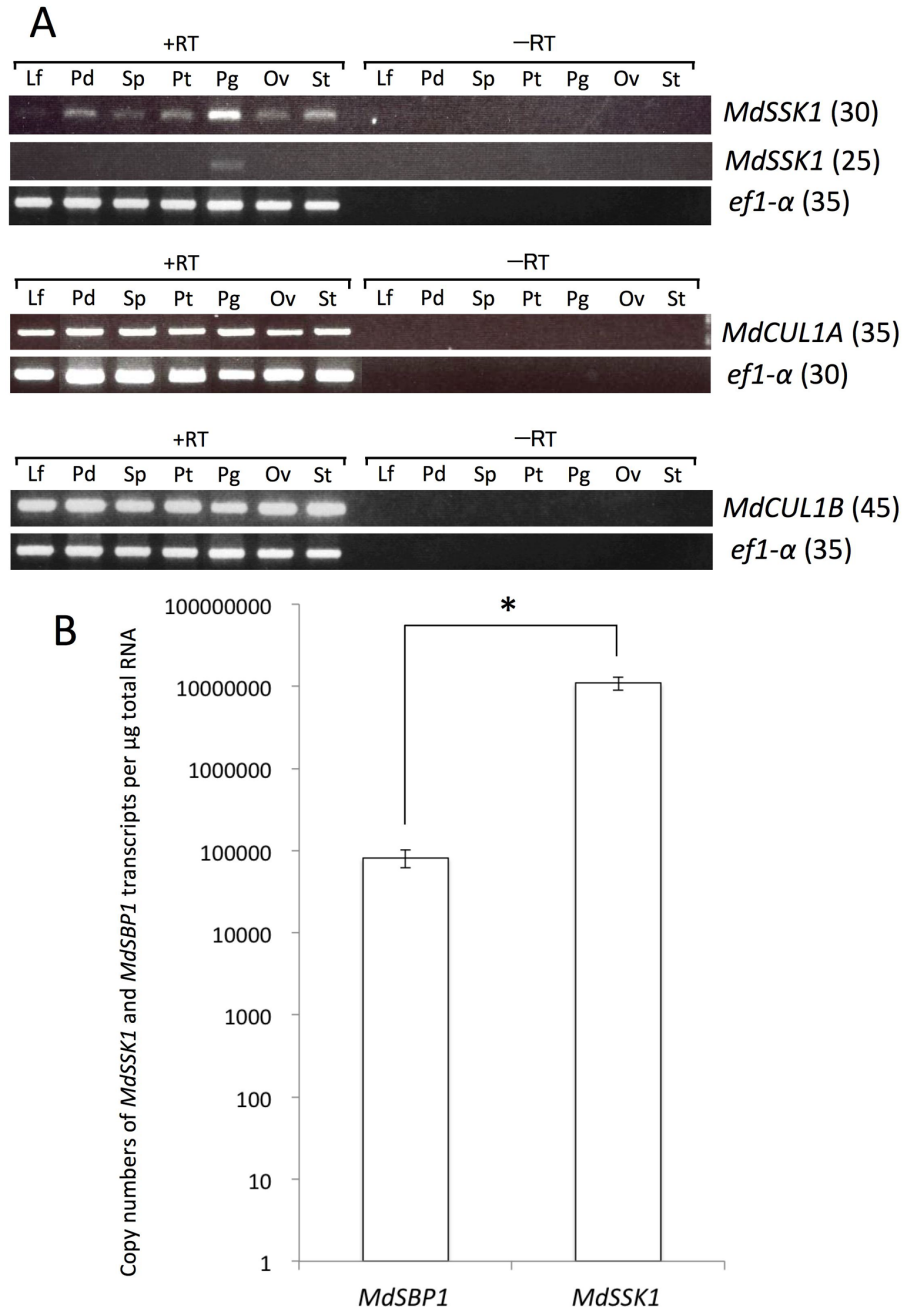
Expression analyses of *MdSSK1*, *MdSBP1* and *MdCUL1s*. (A) RT-PCR analysis of the expression of *MdSSK1* and *MdCUL1s*. *MdSSK1* and *MdCUL1s* were amplified by RT-PCR. The PCR cycle numbers are given in parentheses. RT, reverse transcriptase; Lf, leaf; Pd, pedicel; Sp, sepal; Pt, petal; Pg, pollen grain; Ov, ovary; St, style. (B) Transcript abundances of *MdSSK1* and *MdSBP1* in apple pollen. The mRNA copy numbers per µg total RNA of *MdSSK1* and *MdSBP1* were determined by qRT-PCR, respectively. Mean ± SE of three biological replicates are shown. *, Mean is significantly different (P<0.05) by Student’s *t* test.

RT-PCR analysis revealed that *MdSSK1* was preferentially expressed in pollen (Figure 3A). Using 25 cycles of PCR amplification, *MdSSK1* seemed to be specifically expressed in pollen, and with 30 cycles, signals of *MdSSK1* were observed strongly in pollen, but weakly in other organs analyzed. RT-PCR analysis showed that *MdCUL1s* were expressed in all organs analyzed (Figure 3A). To compare the expression levels of *MdSSK1* and *MdSBP1* in pollen, absolute qRT-PCR was performed. The result showed that *MdSSK1* transcript abundance was significantly (>100 times) higher than that of *MdSBP1* (P<0.05) (Figure 3B).

### Interactions of MdSFBB1*-S* ^9^ with MdSSK1 and MdSBP1

**Figure 4:**
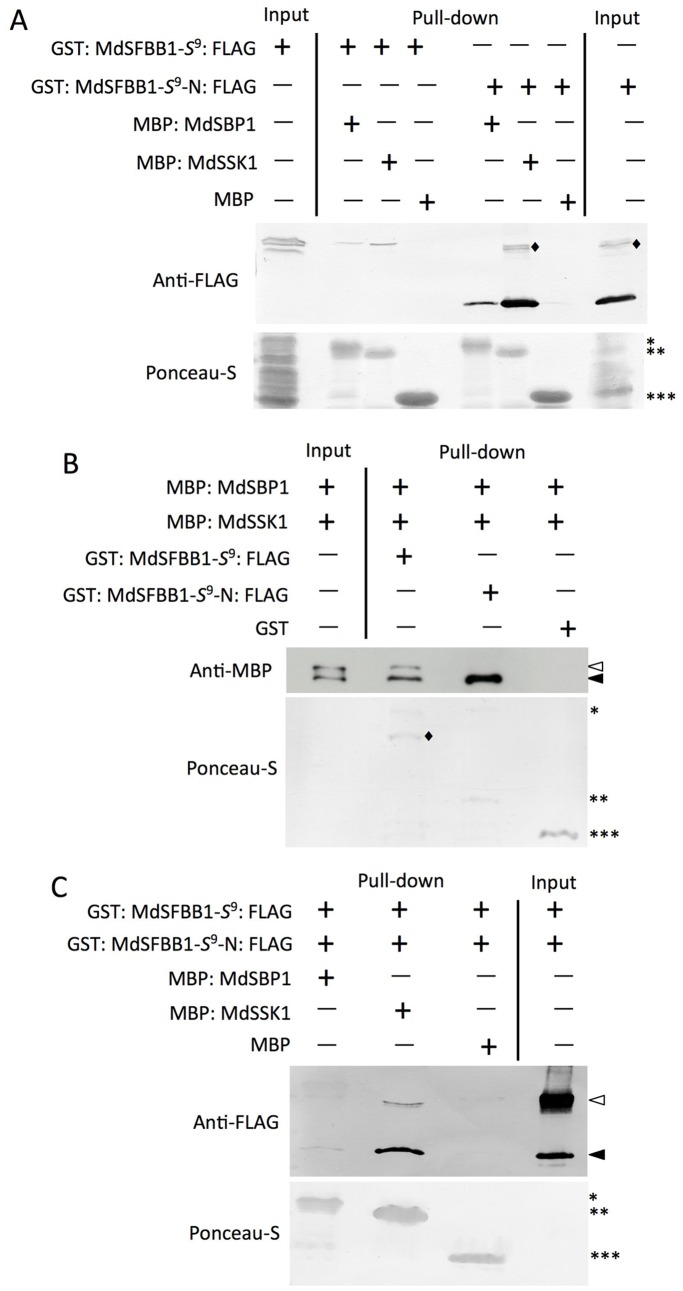
*In vitro* binding assays of MdSSK1 and MdSBP1 with MdSFBB1-*S* ^9^ and MdSFBB1-*S* ^9^-N. (A) Interactions of MdSSK1 and MdSBP1 with MdSFBB1-*S* ^9^ and MdSFBB1-*S* ^9^-N. MBP: MdSSK1, MBP: MdSBP1 and MBP (negative control) were reacted with amylose resin. These beads bound recombinant proteins were incubated with GST: MdSFBB1-*S* ^9^: FLAG and GST: MdSFBB1-*S* ^9^-N: FLAG. Eluted proteins were separated by SDS-PAGE and detected using an anti-FLAG antibody (top). Protein loading was checked by Ponceau-S staining of the blot before immunoblotting (bottom). Single, double and triple asterisks, indicate MBP: MdSBP1, MBP: MdSSK1 and MBP, respectively. Diamonds indicate non-specific signals. (B) Competitive pull-down assay of MdSFBB1-*S* ^9^ and MdSFBB1-*S* ^9^-N with MdSSK1 and MdSBP1. GST: MdSFBB1-*S* ^9^: FLAG, GST: MdSFBB1-*S* ^9^-N: FLAG and GST (negative control) were reacted with Glutathione Sepharose 4B. These sepharose bound recombinant proteins were incubated with an equal amount protein mixture of MBP: MdSSK1 (15 µg) and MBP: MdSBP1 (15 µg). Eluted proteins were separated by SDS-PAGE and detected using an anti-MBP antibody (top). Protein loading was checked by Ponceau-S staining of the blot before immunoblotting (bottom). Single, double and triple asterisks, indicate GST: MdSFBB1-*S* ^9^: FLAG, GST: MdSFBB1-*S* ^9^-N: FLAG and GST, respectively. Opened and closed arrows indicate MBP: MdSBP1 and MBP: MdSSK1, respectively. Diamonds indicate the probable truncated GST: MdSFBB1-*S* ^9^: FLAG. (C) Competitive pull-down assay of MdSSK1 and MdSBP1 with MdSFBB1-*S* ^9^ and MdSFBB1-*S* ^9^-N. MBP: MdSSK1, MBP: MdSBP1 and MBP (negative control) were reacted with amylose resin. These beads bound recombinant proteins were incubated with a protein mixture of approximately equal molecular numbers of GST: MdSFBB1-*S* ^9^: FLAG (74 kDa, 4.5 µg) and GST: MdSFBB1-*S* ^9^-N: FLAG (35 kDa, 2.1 µg). Eluted proteins were separated by SDS-PAGE and detected using an anti-FLAG antibody (top). Protein loading was checked by Ponceau-S staining of the blot before immunoblotting (bottom). Single, double and triple asterisks, indicate MBP: MdSBP1, MBP: MdSSK1 and MBP, respectively. Opened and closed triangles indicate specific GST: MdSFBB1-*S* ^9^: FLAG and GST: MdSFBB1-*S* ^9^-N: FLAG signals, respectively.

We examined the interaction of MdSFBB1-*S* ^9^ with MdSSK1 and MdSBP1 using an *in vitro* binding assay. In the tribe Pyreae of Rosaceae, many pollen *S* candidate genes (*SFBB*) were identified [13]–[17], [36]. Among *SFBB* genes, the *Pyrus pyrifolia SFBB1*-*S* ^4^ (*PpSFBB1*-*S* ^4^/*S* ^4^ *F-box0*) gene was most strongly supported as a pollen *S* by mutant analysis. *S* ^4sm^ pollen lacking *PpSFBB1*-*S* ^4^ of a Japanese pear mutant ‘Osa-Nijisseiki’ [37] was rejected by the pistil harboring not only the self *S* ^4^ but also the non-self *S* ^1^ haplotype, suggesting that *PpSFBB1*-*S* ^4^ would be required for degradation of non-self *S* ^1^-RNase [16], [19]. Because MdSFBB1-*S* ^9^ is a probable ortholog of PpSFBB1-*S* ^4^, we used MdSFBB1-*S* ^9^ for protein-protein interaction analyses. It was reported that the interaction between F-box protein and Skp1 of the SCF complex is mediated through the F-box motif of the F-box protein [38]; therefore, we used the part of the protein (amino acid residues 1–61), N-terminal region containing F-box motif of MdSFBB1-*S* ^9^ named MdSFBB1-*S* ^9^-N, for the binding assay in addition to full-length MdSFBB1-*S* ^9^. MBP-fused MdSSK1 (MBP: MdSSK1), MBP-fused MdSBP1 (MBP: MdSBP1) and MBP (negative control) proteins were expressed in *E. coli* and reacted with amylose resin. The recombinant protein-bound beads were then incubated with a crude extract of *E. coli* expressing GST-fused and FLAG-tagged MdSFBB1-*S* ^9^ (GST: MdSFBB1-*S* ^9^: FLAG) or GST-fused MdSFBB1-*S* ^9^-N (GST: MdSFBB1-*S* ^9^-N: FLAG). Eluted proteins were separated by SDS-PAGE and detected using anti-FLAG antibody. The results showed that both MdSSK1 and MdSBP1 interact with MdSFBB1-*S* ^9^ and MdSFBB1-*S* ^9^-N (Figure 4A). Because MdSFBB1-*S* ^9^ and MdSFBB1-*S* ^9^-N seemed to interact more strongly with MdSSK1 than with MdSBP1, a competitive pull-down assay between the recombinant proteins was conducted. GST: MdSFBB1-*S* ^9^: FLAG, GST: MdSFBB1-*S* ^9^-N: FLAG and GST (negative control) proteins were reacted with Glutathione Sepharose 4B and incubated with a protein mixture of MBP: MdSSK1 and MBP: MdSBP1. The result revealed that MdSSK1 exhibits a stronger interaction affinity to MdSFBB1-*S* ^9^ and MdSFBB1-*S* ^9^-N than MdSBP1 (Figure 4B). Because MdSFBB1-*S* ^9^-N seemed to interact more strongly with MdSSK1 and MdSBP1 than MdSFBB1-*S* ^9^, this possibility was examined by a competitive pull-down assay. MBP: MdSSK1, MBP: MdSBP1 and MBP (negative control) proteins were reacted with amylose resin and incubated with an equal amount protein mixture of GST: MdSFBB1-*S* ^9^: FLAG and GST: MdSFBB1-*S* ^9^-N: FLAG. Taken into account the calculated molecular mass of GST: MdSFBB1-*S* ^9^: FLAG and GST: MdSFBB1-*S* ^9^-N: FLAG, 74 kDa and 35 kDa, respectively, 4.5 µg of GST: MdSFBB1-*S* ^9^: FLAG and 2.1 µg of GST: MdSFBB1-*S* ^9^-N: FLAG were used. The result showed that MdSFBB1-*S* ^9^-N had higher affinity than MdSFBB1-*S* ^9^ for binding to MdSSK1 and MdSBP1 (Figure 4C).

### Interactions of MdCUL1s with MdSSK1 and MdSBP1

**Figure 5:**
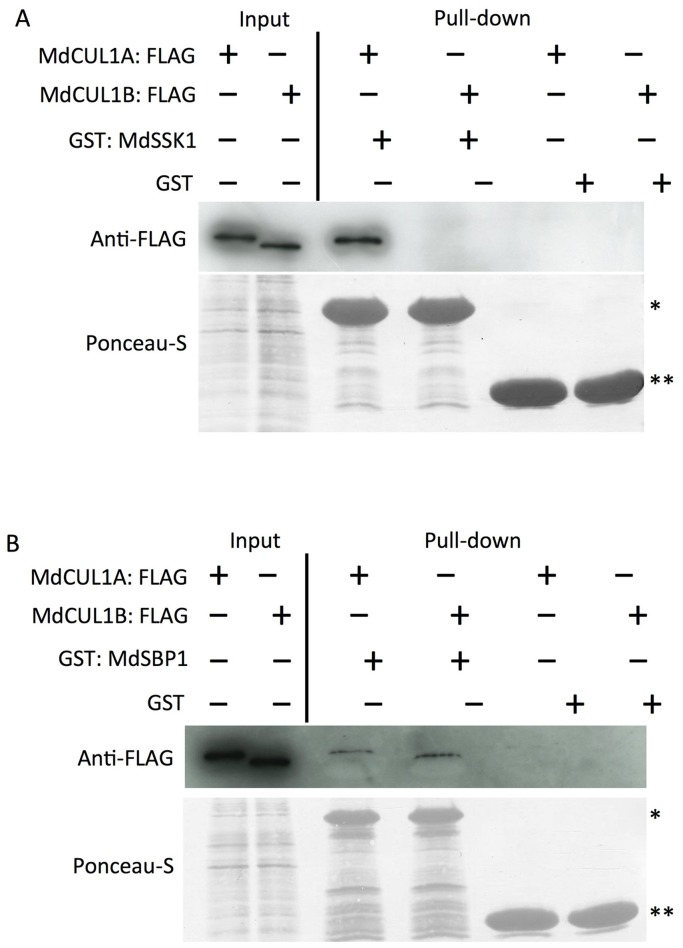
*In vitro* binding assays of MdSSK1 and MdSBP1 with MdCUL1s. The interactions of MdCUL1s with MdSSK1 (A) and MdSBP1 (B) were tested. GST: MdSSK1, GST: MdSBP1 and GST (negative control) were expressed in *E. coli* and reacted with Glutathione Sepharose 4B. These sepharose bound recombinant proteins were incubated with MdCUL1A: FLAG and MdCUL1B: FLAG expressed in a cell-free system. Eluted proteins were separated by SDS-PAGE and detected by using an anti-FLAG antibody (top). Protein loading was checked by Ponceau-S staining of the blot before immunoblotting (bottom). Single and double asterisks indicate the GST-fusion protein and GST, respectively.

To examine the binding of MdCUL1s with MdSSK1 and MdSBP1, *in vitro* binding assays were conducted. GST: MdSSK1, GST: MdSBP1 and GST (negative control) proteins were reacted with Glutathione Sepharose 4B. MdCUL1A: FLAG and MdCUL1B: FLAG proteins were expressed using wheat germ extracts and incubated with the protein-bound Glutathione Sepharose 4B. The results showed that MdSSK1 interacts with MdCUL1A, but not with MdCUL1B, whereas, MdSBP1 interacted with both MdCUL1A and MdCUL1B (Figure 5A, B).

## Discussion

### MdSSK1 and MdSBP1 may form Canonical and Noncanonical SCF Complexes, Respectively, with MdSFBB1 and MdCUL1 in Pollen of Apple

We isolated an *SSK1* homolog (*MdSSK1*) of apple. *MdSSK1* was preferentially expressed in pollen, but weakly in other organs analyzed, whereas, *MdSBP1* was almost equally expressed in all organs analyzed [31]. The expression pattern of *MdSBP1* was the same as Solanaceous *SBP1* homologs [29], [39]–[41], suggesting that MdSBP1 may also be involved in general cellular functions besides pollination [31]. The expression pattern of *MdSSK1* was slightly different from other *SSK1* genes of Rosaceae, Solanaceae and Plantaginaceae. The *SSK1* genes of *Pyrus bretschneideri* of Rosaceae, *Petunia hybrida* and *Petunia inflata* of Solanaceae and *Antirrhinum hispanicum* of Plantaginaceae were reported to show pollen or anther-specific expression patterns [24], [25], [27], [42]. In *Prunus* of Rosaceae, *PavSSK1* was expressed strongly in pollen and anthers, but weakly in styles, suggesting that PavSSK1 serves as an adaptor for not only PavSFB but also PavSLFL1 expressed in pollen, anthers and styles [26]. The expression pattern of *MdSSK1* suggests that MdSSK1 mainly, but not exclusively, functions in pollen.

*In vitro* binding assays revealed that both MdSSK1 and MdSBP1 interact with MdSFBB1-*S* ^9^ and MdSFBB1-*S* ^9^-N. MdSSK1 and MdSBP1 interacted more strongly with MdSFBB1-*S* ^9^-N than with MdSFBB1-*S* ^9^. Given that MdSFBB1-*S* ^9^-N almost corresponds to the F-box domain, this is possibly because MdSSK1 and MdSBP1 interact with MdSFBB1*-S* ^9^ through the F-box domain of MdSFBB1-*S* ^9^, and the conformation of bacterially expressed MdSFBB1-*S* ^9^ was different from that of the native state, affecting binding of the F-box motif with SSK and SBP1. The interaction between the F-box protein and Skp1 of the SCF complex is known to be mediated through the F-box motif [38]. The finding that a truncated *SLF* of *Petunia inflata* (*PiSLF*_2_) without the F-box domain expressed in *S* ^2^ *S* ^3^ plants did not cause breakdown of SI in *S* ^3^ pollen [42], whereas, the full-length *PiSLF*_2_ did [43], suggests that the F-box domain of PiSLF_2_ is required for GSI [42]. The F-box domain of PiSLF_2_ was reported to interact with PiSBP1 in a yeast two-hybrid assay [42]. These findings are consistent with our data in apple proteins that the F-box domain of MdSFBB1-*S* ^9^ may be important for binding to MdSSK1 and MdSBP1.

MdSSK1 interacted with MdCUL1A but not with MdCUL1B, whereas, MdSBP1 interacted with both MdCUL1A and MdCUL1B by *in vitro* binding assays. The N-terminal domain (NTD) of human CUL1 is reported to bind the human Skp1 [33]. The C-terminal domains (CTDs) of MdCUL1A and MdCUL1B are fairly well conserved, however, their NTDs are very different (Figure S4). Divergence at the NTDs of MdCUL1A and MdCUL1B may be responsible for the difference in the affinities of the proteins with MdSSK1. A pollen-expressed *CUL1* gene of *Solanum* is considered to be involved in unilateral interspecific incompatibility (UI) and SI [44], [45]. Generally, UI occurs in crosses between self-incompatible (SI) and self-compatible (SC) species. Pollen of SI species are compatible with the SC pistil, but not *vice versa* (SI *×* SC rule) [46]. The pollen of SI species of *Solanum* express functional *CUL1* genes, whereas, SC species shared the same loss-of-function mutations, even though the pollen fertilities of SC species are normal [44]. *CUL1*-reduced pollen of transgenic plants obtained by introducing *LAT52-CUL1-RNAi* to SI *S. arcanum* was selectively eliminated on non-transgenic SI pistils, but it was not rejected on S-RNase-deficient SC pistils [45]. The results suggest that the functions of *CUL1* genes of *Solanum* species might have diverged evolutionarily, and SI species of *Solanum* shared CUL1 specialized for degradation of S-RNases in addition to other CUL1 homologs for general functions. Our finding that the interaction partner(s) of MdCUL1A and MdCUL1B are different may also reflect the functional divergence of the two CUL1 proteins.

### Putative Functions of MdSSK1-containing SCF^SFBB^ and MdSBP1-containing SCF^SFBB^-like Complexes in Apple

The results of protein-protein interaction analyses suggest that MdSSK1 and MdSBP1 form canonical and noncanonical SCF-like complexes, respectively, with MdSFBB1-*S* ^9^ and MdCUL1 within pollen of apple; however, the functions of the two SCF^SFBB1^(-like) complexes in GSI of apple are unclear at present. In *Petunia inflata*, PiSBP1 interacted with PiSLF1-*S*_2_ and PiCUL1-G, suggesting that PiSBP1 would be a component of a noncanonical E3 ligase complex, which interacts with non-self S-RNases to ubiquitinate them for degradation [29]. Structural similarity of PiSBP1 and the apple homolog MdSBP1 [31], together with the results of *in vitro* binding assays of this study, may suggest that MdSBP1 forms noncanonical SCF complex like PiSBP1. In *Petunia hybrida*, functional analyses of PhSSK1 using RNAi plants (*LAT52-PhSSK1-RNAi*) revealed that a substantial reduction of PhSSK1 in transgenic pollen reduced cross-pollen compatibility, although the transgenic plants retained SI [25], suggesting that the PhSSK1-containing SCF complex is involved in degradation of non-self S-RNases. MdSSK1- and MdSBP1-containing SCF^SFBB1^ (-like) complexes may also be involved in the degradation of non-self S-RNases in pollen of apple. *In vitro* binding assays showed that MdSFBB1-*S* ^9^ and MdSFBB1-*S* ^9^-N interacted more strongly with MdSSK1 than with MdSBP1, suggesting that, in apple pollen, MdSSK1-containing SCF^SFBB^ complexes plays a major role, which is likely to be in the degradation of non-self S-RNases. The higher abundance of *MdSSK1* transcripts than *MdSBP1* transcripts would also support the idea. Recent co-immunoprecipitation assays using pollen extracts of *Petunia inflata* detected PiSSK1 but not PiSBP1 as the co-purified protein with PiSLF [28]. Like in the case of apple, this is possibly because PiSSK1 have higher affinity than PiSBP1 for binding to PiSLF, and/or PiSSK1 is more abundant than PiSBP1 in pollen.

Recent studies provide evidence that SBP1 may have other functions besides self/non-self discrimination in S-RNase-based GSI. SBP1 of *Petunia hybrida,* PhSBP1, could be a candidate for the non-allele-specific inhibitor of all S-RNases because it showed no polymorphism in different *S* alleles [30], [39]. SBP1 of *Nicotiana alata,* NaSBP1, was reported to interact with the C-terminal domain of pistil arabinogalactan proteins (AGPs), transmitting tract-specific glycoprotein (TTS) and 120-kDa glycoprotein (120K), suggesting that binding between NaSBP1 and the pistil AGPs may contribute to signaling and trafficking processes inside pollen tubes [41]. *MdSBP1* and solanaceous *SBP1* homologs were expressed in all tissues examined, and the proteins included the same protein-protein interaction domains, RING-HC finger motif and coiled-coil region [31], suggesting that MdSBP1 may also function besides self/non-self discrimination like as SBP1 homologs of Solanaceae.

In S-RNase-based GSI, two different systems are proposed, ‘collaborative non-self recognition system by multiple factors’ for Solanaceae [18] and rosaceous tribe Pyreae [16], [19], and ‘self recognition system by a single factor’ for *Prunus* of Rosaceae [16], [20], [21]. The ‘collaborative non-self recognition system by multiple factors’ is consistent with ‘competitive interaction (CI)’ known to be a phenomenon that coexistence of different pollen *S* alleles in a pollen grain causes breakdown of pollen SI function [43], [47]. For example, a tetraploid plant with *S^1^S^1^S^2^S^2^* genotype produces three *S*-genotypes of pollen (*S* ^1^ *S* ^1^, *S* ^2^ *S* ^2^ and *S* ^1^ *S* ^2^ *),* and *S* ^1^ *S* ^1^ and *S* ^2^ *S* ^2^ pollen are rejected by the self pistil, but *S* ^1^ *S* ^2^ pollen is accepted. In *S* ^1^ *S* ^2^ heteroallelic pollen, pollen *S* ^1^ and *S* ^2^ proteins would target non-self S^2^-RNase and S^1^-RNase, respectively. The CI phenomenon was reported in the tribe Pyreae of Rosaceae, *Petunia* of Solanaceae and *Antirrhinum* of Plantaginaceae, but not in *Prunus* of Rosaceae [6], [43], [48]–[52]. In *Prunus,* most pollen-part self-compatibility (SC) mutants encode a truncated SFB protein or lack the *SFB* gene [20], [21], [53]–[56]. These findings suggest that the species of *Prunus* exhibit the ‘self recognition system by a single factor’ [16], [20], [21]. This model postulates that, in *Prunus,* non-self S-RNase is inactivated by an unidentified ‘general inhibitor’, while self S-RNase is protected by SFB. The protected self S-RNase would degrade RNA in a self pollen tube to prevent growth. It seems that the pollen *S* functions of the tribe Pyreae and *Prunus* of Rosaceae are different, although SSK1 homologs of the tribe Pyreae and *Prunus* are suggested to form similar SCF^SFBB/SFB^ complexes [26], [27]. Further biochemical characterization and comparative analyses of the functions of SSK1- and SBP1-containing SCF(-like) complexes in S-RNase-based GSI plants would shed light on the difference in the two self/non-self recognition systems of S-RNase-based GSI.

## Materials and Methods

### Plant Materials

Leaves and floral organs of apple cultivar ‘Kitaro’ (*S* ^3^ *S* ^9^) were collected in spring, frozen in liquid nitrogen, and stored at −80°C until use.

### Isolation of cDNA Sequences

RNA was isolated from the leaves and floral organs of apple as described by [10]. Total RNA samples were treated with DNaseI (Nippongene). cDNA was synthesized from the treated RNA as described by [10], and used for RT-PCR.

A full-length coding sequence (*MdSSK1*) homologous to *PavSSK1* [26] was selected from the apple genome database for Rosaceae (http://www.rosaceae.org), and the sequence was amplified using the primer pair MdSkp1r2 (5′-TGAATCCATCCGAAAACGAC-3′) and MdSkp1f2 (5′-GTAACTTTCCTTCAAATTATATAT-3′) with pollen cDNA from apple as a template. The DDBJ/GenBank/EMBL accession number of *MdSSK1* is AB898683.

Two different cDNA sequences (*MdCUL1A* and *MdCUL1B*) homologous to solanaceous *CUL1* were selected from the apple genome database for Rosaceae and the primers were designed. The full-length coding sequences were amplified using the primers FMdcul1 (5′-ATTGTAGTGGGASGGTAGCG-3′) and RMdcul1Sl (5′-TGACGTCGACGGCAAG ATACTTGAACATG-3′) for the first sequence (*MdCUL1A*), and MDP0000302895utr (5′-CCTCACAATTCTCCGGCAG-3′) and MDP0000302895utf (5′-TATATCAAGAGTCAAAGACTCG-3′) for the second (*MdCUL1B*) with pollen cDNA of apple as a template. The DDBJ/GenBank/EMBL accession numbers of *MdCUL1A* and *MdCUL1B* are AB898684 and AB898685, respectively.

The amino acid identities among SSK1 or CUL1 proteins were analyzed using GENETYX-MAC (version 17; Genetyx). The amino acid sequences of SSK1 or CUL1 proteins were aligned using Clustal W [57]. A neighbor-joining tree was constructed [58] based on the alignment using MEGA ver. 5.05. [59].

### RT-PCR and Quantitative Real-time PCR (qRT-PCR)

The expression levels of *MdSSK1, MdCUL1A* and *MdCUL1B* were analyzed by RT-PCR with gene-specific primers for *MdSSK1* (MdPpSkp1Bamf: 5′-CGGGATCCATGTCGACCGAGGAGAAGA-3′ and MdPpSkp1EcoRIr: 5′-CGGAATTCTCAGTCTTCATCGACTCCTT-3′), *MdCUL1A* (FMdcul1Bm: 5′-CGCGGATCCATGGAGCGCAAAGTTATTGAG-3′ and RMdcul1Sl: 5′-TGACGTCGACTCAGGCAAGATACTTGAACATG-3′) and *MdCUL1B* (MDP0000302895utr: 5′-CCTCACAATTCTCCGGCAG-3′ and MdCUL1Bmidr: 5′-ATGGCAGCACCGTTGTGGTTACA-3′). *Ef1-α* used as a control was amplified using primers ef1-αf2 (5′-ATTGTGGTCATTGGYCAYGT-3′) and ef1-αr1 (5′-CCTATCTTGTAVACATCCTG-3′).

Transcript abundances of *MdSSK1* and *MdSBP1* in pollen were measured by qRT-PCR with KOD SYBR qPCR Mix (Toyobo) using each gene specific primers for MdSSK1 (MdSSK1qPCRf1∶5′- AGTTCCAGACCGTCAAGTC -3′ and MdSSK1qPCRr1∶5′-TTAACTCCCTGACGAAATCG-3′), and MdSBP1(MdSBP1qPCRf1∶5′-CGGATGGCAGCGATAATTG-3′ and MdSBP1qPCRr1∶5′-CCTGTAGAAGCCATTCGAG-3′). Data were collected using ABI PRISM 7000 sequence detection system (Applied Biosystems) in accordance with the instruction manual. The cDNA sequences of the two genes were cloned into vector pEU3-NII (Toyobo). The plasmid DNA containing the two genes was used to generate standard curves for absolute quantification. *C*_T_ values for each sample were converted into absolute copy numbers (x) using the standard curves (x = (y intercept - *C*_T_)/slope).

### Construction of Plasmids

The full-length coding sequence of *MdSFBB1*-*S* ^9^ [14], [16] and the partial cDNA sequence for the N-terminal region containing the F-box motif of *MdSFBB1*-*S* ^9^ named *MdSFBB1-S* ^9^ *-N* were amplified using primers for *MdSFBB1*-*S* ^9^ (MdFBX16Bmf2∶5′-CGGGATCCATGTTCCAGGTGCGTGAAAGT-3′ and MdFBX16nostpSper: 5′-CGGACTAGTCTTGACTGGAACAATACTTTC-3′) and for *MdSFBB1-S* ^9^ *-N* (MdFBX16Bmf2∶5′-CGGGATCCATGTTCCAGGTGCGTGAAAGT-3′ and MdFBX16motifSper: 5′-CGGACTAGTGGATGATGATAGTTTGTTGTC-3′) from an apple BAC 21M5 [14]. The coding sequence of glutathione S-transferase (GST) was cloned into vector pColdIV (Takara Bio) to produce pColdIVGST. The *Bam*HI-*Spe*I fragment of *MdSFBB1*-*S* ^9^ or *MdSFBB1-S* ^9^ *-N* and a *Spe*I-*Hin*dIII fragment for FLAG tag (DYKDDDDK) were then cloned into the *Bam*HI and *Hin*dIII sites of pColdIVGST to produce pColdGSTMdSFBB1-*S* ^9^FLAG and pColdGSTMdSFBB1-*S* ^9^-NFLAG for expression of GST-fused and FLAG-tagged MdSFBB1-*S* ^9^ and MdSFBB1*-S* ^9^ *-*N proteins (GST: MdSFBB1-*S* ^9^: FLAG and GST: MdSFBB1-*S* ^9^-N: FLAG), respectively.

The full-length coding sequence of *MdSSK1* was amplified using primers MdPpSkp1Bamf (5′-CGGGATCCATGTCGACCGAGGAGAAGA-3′) and MdPpSkp1EcoRIr (5′-CGGAATTCTCAGTCTTCATCGACTCCTT-3′) and was then cloned into vector pColdIVGST at the *Bam*HI and *Eco*RI sites to produce pColdGSTMdSSK1 for the expression of GST-fused MdSSK1 protein (GST: MdSSK1). To produce maltose binding protein (MBP)-fused MdSSK1 protein (MBP: MdSSK1), the *Bam*HI-*Xba*I fragment of *MdSSK1* released from the pColdGSTMdSSK1 construct was cloned into vector pColdIIMBP [31] (pColdMBPMdSSK1).

To produce MBP-fused MdSBP1 protein, the full-length coding sequence of *MdSBP1* was cloned into vector pColdIIMBP [31] to construct pColdMBPMdSBP1. The *Bam*HI-*Xba*I fragment of *MdSBP1* released from the pColdMBPMdSBP1 construct was cloned into vector pColdIVGST to make pColdGSTMdSBP1 for expression of GST-fused MdSBP1 protein (GST: MdSBP1).

The full-length coding sequences of *MdCUL1A* and *MdCUL1B* were amplified by PCR using primers; FMdcul1Xba (5′-GCTCTAGAATGGAGCGCAAAGTTATTGAG-3′) and RMdcul1NostpSpe (5′- CGGACTAGTGGCAAGATACTTGAACATGTTG-3′) for *MdCUL1A*, and XbaEcoVMDP302895f (5′-GCTCTAGAGATATCATGAGTGTGGACTCAGGTAG-3′) and XbaMDP302895nostpr (5′-GCTCTAGACGCAAGATACTTAAACAAGTTAC-3′) for *MdCUL1B*. The *Xba*I-*Spe*I fragment of *MdCUL1A* and a *Spe*I-*Bam*HI fragment of the coding sequence of FLAG tag were cloned into the *Spe*I and *Bam*HI sites of vector pEU3-NII (Toyobo) (pEUMdCUL1AFLAG) for expression of FLAG-tagged MdCUL1A protein (MdCUL1A: FLAG). To produce FLAG-tagged MdCUL1B protein (MdCUL1B: FLAG), the coding sequence of *MdCUL1A* released from pEUMdCUL1AFLAG was replaced with that of *MdCUL1B* (pEUMdCUL1BFLAG).

### Pull-down Assays

Constructs, except for pEU3MdCUL1AFLAG and pEU3MdCUL1BFLAG, were introduced into BL21 (DE3) pLysS (Novagen) and cultured and induced as described in [31]. pColdIIMBP and pColdIVGST were also transferred to BL21 (DE3) pLysS for the expression of MBP and GST, respectively, as negative controls in the pull-down assay. MBP: MdSSK1, MBP: MdSBP1 and MBP were extracted from bacteria by sonication, and reacted with amylose resin (New England BioLabs) in binding buffer [31]. Crude proteins of GST: MdSFBB1*-S* ^9^: FLAG and GST: MdSFBB1*-S* ^9^-N: FLAG were extracted from bacteria and incubated with protein-bound amylose resin at 4°C for 2 hours. For a competitive pull-down assay of MdSSK1 and MdSBP1 with MdSFBB1*-S* ^9^ and MdSFBB1*-S* ^9^-N, a recombinant protein mixture of GST: MdSFBB1*-S* ^9^: FLAG and GST: MdSFBB1*-S* ^9^-N: FLAG were incubated with protein-bound amylose resin. Taken into account the calculated molecular mass of GST: MdSFBB1-*S* ^9^: FLAG and GST: MdSFBB1-*S* ^9^-N: FLAG, 74 kDa and 35 kDa, respectively, 4.5 µg of GST: MdSFBB1-*S* ^9^: FLAG and 2.1 µg of GST: MdSFBB1-*S* ^9^-N: FLAG were used. The beads were washed five times with washing buffer [31], and the proteins were eluted from the beads using maltose-containing native elution buffer (20 mM Tris-HCl pH 7.5, 0.2 M NaCl, 1 mM EDTA, 10 mM maltose). The eluted proteins were separated by SDS-PAGE and detected using an anti-FLAG M2 monoclonal antibody (SIGMA).

For the next competitive pull-down assay of MdSFBB1*-S* ^9^ and MdSFBB1*-S* ^9^-N with MdSSK1 and MdSBP1, GST: MdSFBB1*-S* ^9^: FLAG and GST: MdSFBB1*-S* ^9^-N: FLAG were reacted with Glutathione Sepharose 4B (GE Healthcare). Equal amounts of recombinant protein mixture of MBP: MdSSK1 (15 µg) and MBP: MdSBP1 (15 µg) were incubated with protein-bound Glutathione Sepharose 4B. The beads were washed five times with washing buffer [31], and the proteins were eluted from the beads using glutathione-containing native elution buffer (50 mM Tris-HCl pH 8.0, 10 mM reduced glutathione). The eluted proteins were separated by SDS-PAGE and detected using an anti-MBP monoclonal antibody (HRP-conjugated) (New England BioLabs).

pEU3MdCUL1AFLAG and pEU3MdCUL1BFLAG constructs were used for *in vitro* transcription with T7 RNA polymerase. The transcripts were then *in vitro* translated using wheat germ extracts (CellFree Sciences) at 25°C for 24 h. The translation products were used for the pull-down assay. Crude proteins of GST: MdSSK1, GST: MdSBP1 and GST were reacted with Glutathione Sepharose 4B. The MdCUL1A: FLAG and MdCUL1B: FLAG proteins were incubated with protein-bound Glutathione Sepharose 4B at 4°C for 2 hours. The beads were washed five times with washing buffer [31], and the proteins were eluted from the beads using 2 × SDS loading buffer [31]. The eluted proteins were separated by SDS-PAGE and detected using an anti-FLAG M2 monoclonal antibody (SIGMA).

## Supporting Information

Figure S1 Amino acid sequence alignment of MdSSK1 and plant Skp1-like proteins. Amino acid sequences were aligned using Clustal W. MdSSK1 (AB898683), PbSSK1 (CCH26218), PbSSK2 (CCH26217), PavSSK1 (AFJ21661), PavPSK1 (AFJ21662), PiSSK1 (AEE39461), PhSSK1 (ACT35733), AhSSK1 (ABC84199), ASK1 (NP_565123), ASK2 (NP_568603) ASK3 (NP_565604), ASK4 (NP_564105), ASK5 (NP_567091), ASK6 (NP_566978), ASK7 (NP_566693), ASK8 (NP_566692), ASK9 (NP_566694), ASK10 (NP_566695), ASK11 (NP_567959), ASK12 (NP_567967), ASK13 (NP_567090), ASK14 (NP_565296), ASK15 (NP_566773), ASK16 (NP_565297), ASK17 (NP_565467), ASK18 (NP_563864), ASK19 (NP_565295), ASK20 (NP_566058), ASK21 (NP_567113) and OSK1 (LOC_Os11g26910) were from *Malus* × *domestica, Pyrus bretschneideri, Prunus avium, Petunia inflata, Petunia hybrida, Antirrhinum hispanicum, Arabidopsis thaliana* and *Oryza sativa,* respectively. Conserved sites and relatively conservative sites are marked with asterisks and dots, respectively.
(PPTX)

Figure S2 Neighbor-joining tree of MdCUL1s and other plant CUL-like proteins. The tree was constructed based on the aligned deduced amino acid sequences from apple (MdCUL1A, AB898684; MdCUL1B, AB898685), *Pyrus bretschneideri* (PbCUL1, CCH26221), sweet cherry (PavCUL1A, AFJ21664; PavCUL1B, AFJ21665), *Petunia* (PiCUL1G, ABB77429; PiCUL1C, ABB77428; PhCUL1, ACT35735), *Solanum pennellii* (SpCUL1, ADU60534), *Arabidopsis* (AtCUL1, NP_001031575; AtCUL2, NP_171797; AtCUL3A, NP_174005; AtCUL3B, NP_177125; AtCUL4, NP_568658) and rice (OsCUL1-like, LOC_Os01g27150; OsCUL3-like, LOC_Os02g51180; OsCUL4-like, LOC_Os03g57290). The tree was generated with 1000 bootstrap replicates. ScCDC53 (NP_010150) was defined as the outgroup.
(PPTX)

Figure S3 Amino acid sequence alignment of MdCUL1 and other plant CUL-like proteins. Amino acid sequences were aligned using Clustal W. Conserved sites and relatively conservative sites are marked with asterisks and dots, respectively. The NEDD8 domain detected by Pfam (http://pfam.sanger.ac.uk) is denoted by a line. Abbreviations: Md, *Malus × domestica;* Pb, *Pyrus bretschneideri;* Pav, *Prunus avium;* Pi, *Petunia inflata;* Ph, *Petunia hybrida;* Sp, *Solanum pennellii;* At, *Arabidopsis thaliana;* Os, *Oryza sativa* and Sc, *Saccharomyces cerevisiae.* Accession numbers: PbCUL1 (CCH26221), PavCUL1A (AFJ21664), PavCUL1B (AFJ21665), PiCUL1G (ABB77429), PiCUL1C (ABB77428), PhCUL1 (ACT35735), SpCUL1 (ADU60534), AtCUL1 (NP_001031575), AtCUL2 (NP_171797), AtCUL3A (NP_174005), AtCUL3B (NP_177125), AtCUL4 (NP_568658), OsCUL1-like (LOC_Os01g27150), OsCUL3-like (LOC_Os02g51180), OsCUL4-like (LOC_Os03g57290), ScCDC53 (NP_010150).
(PPTX)

Figure S4 Amino acid sequence alignment of MdCUL1A and MdCUL1B. Amino acid sequences were aligned using Clustal W. Conserved sites are marked with asterisks.
(PPTX)

Table S1 Amino acid identities (%) among MdCUL1s and other plant CUL1-like proteins.
(PPTX)
