# Supplementary material for: Identification of SFBB-Containing Canonical and Noncanonical SCF Complexes in Pollen of Apple (Malus × domestica)
Source: PLoS One. 2014 May 21;9(5):e97642. doi: 10.1371/journal.pone.0097642 (PMC4029751; doi:10.1371/journal.pone.0097642)
Supplement: Figure S1 — Amino acid sequence alignment of MdSSK1 and plant Skp1-like proteins. Amino acid sequences were aligned using Clustal W. MdSSK1 (AB898683), PbSSK1 (CCH26218), PbSSK2 (CCH26217), PavSSK1 (AFJ21661), PavPSK1 (AFJ21662), PiSSK1 (AEE39461), PhSSK1 (ACT35733), AhSSK1 (ABC84199), ASK1 (NP_565123), ASK2 (NP_568603) ASK3 (NP_565604), ASK4 (NP_564105), ASK5 (NP_567091), ASK6 (NP_566978), ASK7 (NP_566693), ASK8 (NP_566692), ASK9 (NP_566694), ASK10 (NP_566695), ASK11 (NP_567959), ASK12 (NP_567967), ASK13 (NP_567090), ASK14 (NP_565296), ASK15 (NP_566773), ASK16 (NP_565297), ASK17 (NP_565467), ASK18 (NP_563864), ASK19 (NP_565295), ASK20 (NP_566058), ASK21 (NP_567113) and OSK1 (LOC_Os11g26910) were from Malus × domestica, Pyrus bretschneideri, Prunus avium, Petunia inflata, Petunia hybrida, Antirrhinum hispanicum, Arabidopsis thaliana and Oryza sativa, respectively. Conserved sites and relatively conservative sites are marked with asterisks and dots, respectively. (PPTX) [file pone.0097642.s001.pptx]

## Slide 1
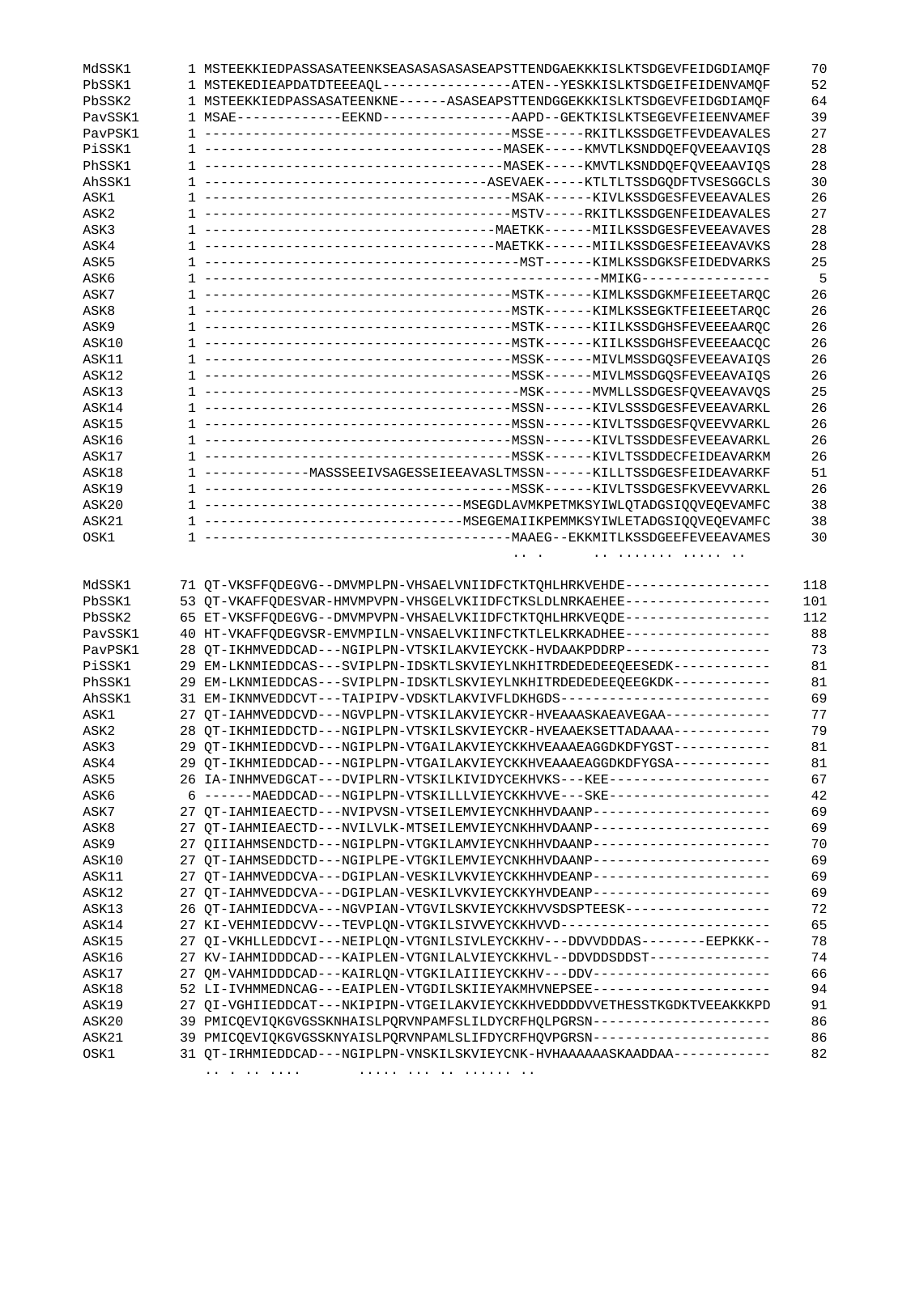

MdSSK1 1 MSTEEKKIEDPASSASATEENKSEASASASASASEAPSTTENDGAEKKKISLKTSDGEVFEIDGDIAMQF 70
PbSSK1 1 MSTEKEDIEAPDATDTEEEAQL----------------ATEN--YESKKISLKTSDGEIFEIDENVAMQF 52
PbSSK2 1 MSTEEKKIEDPASSASATEENKNE------ASASEAPSTTENDGGEKKKISLKTSDGEVFEIDGDIAMQF 64
PavSSK1 1 MSAE-------------EEKND----------------AAPD--GEKTKISLKTSEGEVFEIEENVAMEF 39
PavPSK1 1 --------------------------------------MSSE-----RKITLKSSDGETFEVDEAVALES 27
PiSSK1 1 -------------------------------------MASEK-----KMVTLKSNDDQEFQVEEAAVIQS 28
PhSSK1 1 -------------------------------------MASEK-----KMVTLKSNDDQEFQVEEAAVIQS 28
AhSSK1 1 -----------------------------------ASEVAEK-----KTLTLTSSDGQDFTVSESGGCLS 30
ASK1 1 --------------------------------------MSAK------KIVLKSSDGESFEVEEAVALES 26
ASK2 1 --------------------------------------MSTV-----RKITLKSSDGENFEIDEAVALES 27
ASK3 1 ------------------------------------MAETKK------MIILKSSDGESFEVEEAVAVES 28
ASK4 1 ------------------------------------MAETKK------MIILKSSDGESFEIEEAVAVKS 28
ASK5 1 ---------------------------------------MST------KIMLKSSDGKSFEIDEDVARKS 25
ASK6 1 -------------------------------------------------MMIKG---------------- 5
ASK7 1 --------------------------------------MSTK------KIMLKSSDGKMFEIEEETARQC 26
ASK8 1 --------------------------------------MSTK------KIMLKSSEGKTFEIEEETARQC 26
ASK9 1 --------------------------------------MSTK------KIILKSSDGHSFEVEEEAARQC 26
ASK10 1 --------------------------------------MSTK------KIILKSSDGHSFEVEEEAACQC 26
ASK11 1 --------------------------------------MSSK------MIVLMSSDGQSFEVEEAVAIQS 26
ASK12 1 --------------------------------------MSSK------MIVLMSSDGQSFEVEEAVAIQS 26
ASK13 1 ---------------------------------------MSK------MVMLLSSDGESFQVEEAVAVQS 25
ASK14 1 --------------------------------------MSSN------KIVLSSSDGESFEVEEAVARKL 26
ASK15 1 --------------------------------------MSSN------KIVLTSSDGESFQVEEVVARKL 26
ASK16 1 --------------------------------------MSSN------KIVLTSSDDESFEVEEAVARKL 26
ASK17 1 --------------------------------------MSSK------KIVLTSSDDECFEIDEAVARKM 26
ASK18 1 -------------MASSSEEIVSAGESSEIEEAVASLTMSSN------KILLTSSDGESFEIDEAVARKF 51
ASK19 1 --------------------------------------MSSK------KIVLTSSDGESFKVEEVVARKL 26
ASK20 1 --------------------------------MSEGDLAVMKPETMKSYIWLQTADGSIQQVEQEVAMFC 38
ASK21 1 --------------------------------MSEGEMAIIKPEMMKSYIWLETADGSIQQVEQEVAMFC 38
OSK1 1 --------------------------------------MAAEG--EKKMITLKSSDGEEFEVEEAVAMES 30
 .. . .. ....... ..... ..
MdSSK1 71 QT-VKSFFQDEGVG--DMVMPLPN-VHSAELVNIIDFCTKTQHLHRKVEHDE------------------ 118
PbSSK1 53 QT-VKAFFQDESVAR-HMVMPVPN-VHSGELVKIIDFCTKSLDLNRKAEHEE------------------ 101
PbSSK2 65 ET-VKSFFQDEGVG--DMVMPVPN-VHSAELVKIIDFCTKTQHLHRKVEQDE------------------ 112
PavSSK1 40 HT-VKAFFQDEGVSR-EMVMPILN-VNSAELVKIINFCTKTLELKRKADHEE------------------ 88
PavPSK1 28 QT-IKHMVEDDCAD---NGIPLPN-VTSKILAKVIEYCKK-HVDAAKPDDRP------------------ 73
PiSSK1 29 EM-LKNMIEDDCAS---SVIPLPN-IDSKTLSKVIEYLNKHITRDEDEDEEQEESEDK------------ 81
PhSSK1 29 EM-LKNMIEDDCAS---SVIPLPN-IDSKTLSKVIEYLNKHITRDEDEDEEQEEGKDK------------ 81
AhSSK1 31 EM-IKNMVEDDCVT---TAIPIPV-VDSKTLAKVIVFLDKHGDS-------------------------- 69
ASK1 27 QT-IAHMVEDDCVD---NGVPLPN-VTSKILAKVIEYCKR-HVEAAASKAEAVEGAA------------- 77
ASK2 28 QT-IKHMIEDDCTD---NGIPLPN-VTSKILSKVIEYCKR-HVEAAEKSETTADAAAA------------ 79
ASK3 29 QT-IKHMIEDDCVD---NGIPLPN-VTGAILAKVIEYCKKHVEAAAEAGGDKDFYGST------------ 81
ASK4 29 QT-IKHMIEDDCAD---NGIPLPN-VTGAILAKVIEYCKKHVEAAAEAGGDKDFYGSA------------ 81
ASK5 26 IA-INHMVEDGCAT---DVIPLRN-VTSKILKIVIDYCEKHVKS---KEE-------------------- 67
ASK6 6 ------MAEDDCAD---NGIPLPN-VTSKILLLVIEYCKKHVVE---SKE-------------------- 42
ASK7 27 QT-IAHMIEAECTD---NVIPVSN-VTSEILEMVIEYCNKHHVDAANP---------------------- 69
ASK8 27 QT-IAHMIEAECTD---NVILVLK-MTSEILEMVIEYCNKHHVDAANP---------------------- 69
ASK9 27 QIIIAHMSENDCTD---NGIPLPN-VTGKILAMVIEYCNKHHVDAANP---------------------- 70
ASK10 27 QT-IAHMSEDDCTD---NGIPLPE-VTGKILEMVIEYCNKHHVDAANP---------------------- 69
ASK11 27 QT-IAHMVEDDCVA---DGIPLAN-VESKILVKVIEYCKKHHVDEANP---------------------- 69
ASK12 27 QT-IAHMVEDDCVA---DGIPLAN-VESKILVKVIEYCKKYHVDEANP---------------------- 69
ASK13 26 QT-IAHMIEDDCVA---NGVPIAN-VTGVILSKVIEYCKKHVVSDSPTEESK------------------ 72
ASK14 27 KI-VEHMIEDDCVV---TEVPLQN-VTGKILSIVVEYCKKHVVD-------------------------- 65
ASK15 27 QI-VKHLLEDDCVI---NEIPLQN-VTGNILSIVLEYCKKHV---DDVVDDDAS--------EEPKKK-- 78
ASK16 27 KV-IAHMIDDDCAD---KAIPLEN-VTGNILALVIEYCKKHVL--DDVDDSDDST--------------- 74
ASK17 27 QM-VAHMIDDDCAD---KAIRLQN-VTGKILAIIIEYCKKHV---DDV---------------------- 66
ASK18 52 LI-IVHMMEDNCAG---EAIPLEN-VTGDILSKIIEYAKMHVNEPSEE---------------------- 94
ASK19 27 QI-VGHIIEDDCAT---NKIPIPN-VTGEILAKVIEYCKKHVEDDDDVVETHESSTKGDKTVEEAKKKPD 91
ASK20 39 PMICQEVIQKGVGSSKNHAISLPQRVNPAMFSLILDYCRFHQLPGRSN---------------------- 86
ASK21 39 PMICQEVIQKGVGSSKNYAISLPQRVNPAMLSLIFDYCRFHQVPGRSN---------------------- 86
OSK1 31 QT-IRHMIEDDCAD---NGIPLPN-VNSKILSKVIEYCNK-HVHAAAAAASKAADDAA------------ 82
 .. . .. .... ..... ... .. ...... ..

## Slide 2
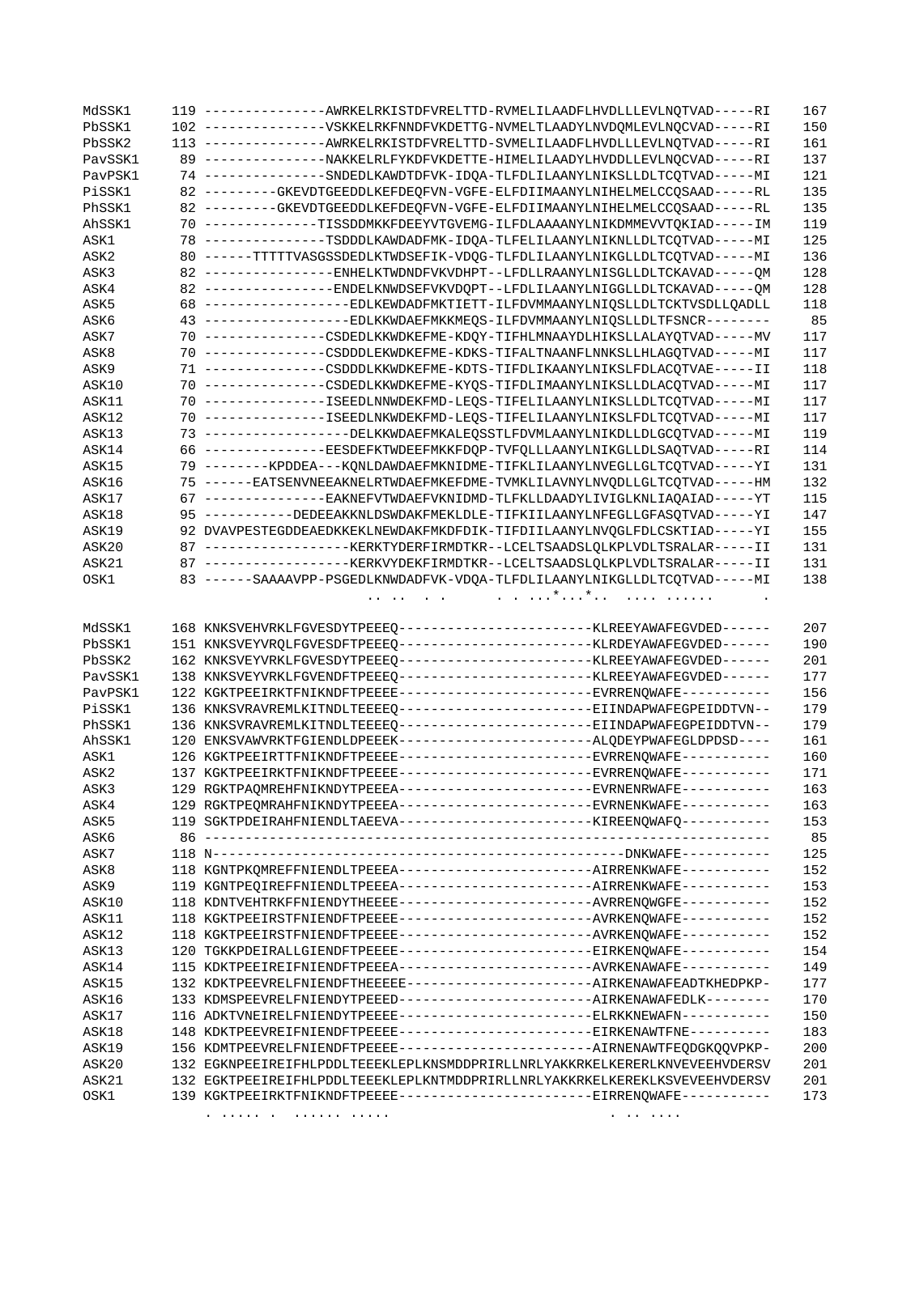

MdSSK1 119 ---------------AWRKELRKISTDFVRELTTD-RVMELILAADFLHVDLLLEVLNQTVAD-----RI 167
PbSSK1 102 ---------------VSKKELRKFNNDFVKDETTG-NVMELTLAADYLNVDQMLEVLNQCVAD-----RI 150
PbSSK2 113 ---------------AWRKELRKISTDFVRELTTD-SVMELILAADFLHVDLLLEVLNQTVAD-----RI 161
PavSSK1 89 ---------------NAKKELRLFYKDFVKDETTE-HIMELILAADYLHVDDLLEVLNQCVAD-----RI 137
PavPSK1 74 ---------------SNDEDLKAWDTDFVK-IDQA-TLFDLILAANYLNIKSLLDLTCQTVAD-----MI 121
PiSSK1 82 ---------GKEVDTGEEDDLKEFDEQFVN-VGFE-ELFDIIMAANYLNIHELMELCCQSAAD-----RL 135
PhSSK1 82 ---------GKEVDTGEEDDLKEFDEQFVN-VGFE-ELFDIIMAANYLNIHELMELCCQSAAD-----RL 135
AhSSK1 70 --------------TISSDDMKKFDEEYVTGVEMG-ILFDLAAAANYLNIKDMMEVVTQKIAD-----IM 119
ASK1 78 ---------------TSDDDLKAWDADFMK-IDQA-TLFELILAANYLNIKNLLDLTCQTVAD-----MI 125
ASK2 80 ------TTTTTVASGSSDEDLKTWDSEFIK-VDQG-TLFDLILAANYLNIKGLLDLTCQTVAD-----MI 136
ASK3 82 ----------------ENHELKTWDNDFVKVDHPT--LFDLLRAANYLNISGLLDLTCKAVAD-----QM 128
ASK4 82 ----------------ENDELKNWDSEFVKVDQPT--LFDLILAANYLNIGGLLDLTCKAVAD-----QM 128
ASK5 68 ------------------EDLKEWDADFMKTIETT-ILFDVMMAANYLNIQSLLDLTCKTVSDLLQADLL 118
ASK6 43 ------------------EDLKKWDAEFMKKMEQS-ILFDVMMAANYLNIQSLLDLTFSNCR-------- 85
ASK7 70 ---------------CSDEDLKKWDKEFME-KDQY-TIFHLMNAAYDLHIKSLLALAYQTVAD-----MV 117
ASK8 70 ---------------CSDDDLEKWDKEFME-KDKS-TIFALTNAANFLNNKSLLHLAGQTVAD-----MI 117
ASK9 71 ---------------CSDDDLKKWDKEFME-KDTS-TIFDLIKAANYLNIKSLFDLACQTVAE-----II 118
ASK10 70 ---------------CSDEDLKKWDKEFME-KYQS-TIFDLIMAANYLNIKSLLDLACQTVAD-----MI 117
ASK11 70 ---------------ISEEDLNNWDEKFMD-LEQS-TIFELILAANYLNIKSLLDLTCQTVAD-----MI 117
ASK12 70 ---------------ISEEDLNKWDEKFMD-LEQS-TIFELILAANYLNIKSLFDLTCQTVAD-----MI 117
ASK13 73 ------------------DELKKWDAEFMKALEQSSTLFDVMLAANYLNIKDLLDLGCQTVAD-----MI 119
ASK14 66 ---------------EESDEFKTWDEEFMKKFDQP-TVFQLLLAANYLNIKGLLDLSAQTVAD-----RI 114
ASK15 79 --------KPDDEA---KQNLDAWDAEFMKNIDME-TIFKLILAANYLNVEGLLGLTCQTVAD-----YI 131
ASK16 75 ------EATSENVNEEAKNELRTWDAEFMKEFDME-TVMKLILAVNYLNVQDLLGLTCQTVAD-----HM 132
ASK17 67 ---------------EAKNEFVTWDAEFVKNIDMD-TLFKLLDAADYLIVIGLKNLIAQAIAD-----YT 115
ASK18 95 -----------DEDEEAKKNLDSWDAKFMEKLDLE-TIFKIILAANYLNFEGLLGFASQTVAD-----YI 147
ASK19 92 DVAVPESTEGDDEAEDKKEKLNEWDAKFMKDFDIK-TIFDIILAANYLNVQGLFDLCSKTIAD-----YI 155
ASK20 87 ------------------KERKTYDERFIRMDTKR--LCELTSAADSLQLKPLVDLTSRALAR-----II 131
ASK21 87 ------------------KERKVYDEKFIRMDTKR--LCELTSAADSLQLKPLVDLTSRALAR-----II 131
OSK1 83 ------SAAAAVPP-PSGEDLKNWDADFVK-VDQA-TLFDLILAANYLNIKGLLDLTCQTVAD-----MI 138
 .. .. . . . . ...*...*.. .... ...... .
MdSSK1 168 KNKSVEHVRKLFGVESDYTPEEEQ------------------------KLREEYAWAFEGVDED------ 207
PbSSK1 151 KNKSVEYVRQLFGVESDFTPEEEQ------------------------KLRDEYAWAFEGVDED------ 190
PbSSK2 162 KNKSVEYVRKLFGVESDYTPEEEQ------------------------KLREEYAWAFEGVDED------ 201
PavSSK1 138 KNKSVEYVRKLFGVENDFTPEEEQ------------------------KLREEYAWAFEGVDED------ 177
PavPSK1 122 KGKTPEEIRKTFNIKNDFTPEEEE------------------------EVRRENQWAFE----------- 156
PiSSK1 136 KNKSVRAVREMLKITNDLTEEEEQ------------------------EIINDAPWAFEGPEIDDTVN-- 179
PhSSK1 136 KNKSVRAVREMLKITNDLTEEEEQ------------------------EIINDAPWAFEGPEIDDTVN-- 179
AhSSK1 120 ENKSVAWVRKTFGIENDLDPEEEK------------------------ALQDEYPWAFEGLDPDSD---- 161
ASK1 126 KGKTPEEIRTTFNIKNDFTPEEEE------------------------EVRRENQWAFE----------- 160
ASK2 137 KGKTPEEIRKTFNIKNDFTPEEEE------------------------EVRRENQWAFE----------- 171
ASK3 129 RGKTPAQMREHFNIKNDYTPEEEA------------------------EVRNENRWAFE----------- 163
ASK4 129 RGKTPEQMRAHFNIKNDYTPEEEA------------------------EVRNENKWAFE----------- 163
ASK5 119 SGKTPDEIRAHFNIENDLTAEEVA------------------------KIREENQWAFQ----------- 153
ASK6 86 ---------------------------------------------------------------------- 85
ASK7 118 N---------------------------------------------------DNKWAFE----------- 125
ASK8 118 KGNTPKQMREFFNIENDLTPEEEA------------------------AIRRENKWAFE----------- 152
ASK9 119 KGNTPEQIREFFNIENDLTPEEEA------------------------AIRRENKWAFE----------- 153
ASK10 118 KDNTVEHTRKFFNIENDYTHEEEE------------------------AVRRENQWGFE----------- 152
ASK11 118 KGKTPEEIRSTFNIENDFTPEEEE------------------------AVRKENQWAFE----------- 152
ASK12 118 KGKTPEEIRSTFNIENDFTPEEEE------------------------AVRKENQWAFE----------- 152
ASK13 120 TGKKPDEIRALLGIENDFTPEEEE------------------------EIRKENQWAFE----------- 154
ASK14 115 KDKTPEEIREIFNIENDFTPEEEA------------------------AVRKENAWAFE----------- 149
ASK15 132 KDKTPEEVRELFNIENDFTHEEEEE-----------------------AIRKENAWAFEADTKHEDPKP- 177
ASK16 133 KDMSPEEVRELFNIENDYTPEEED------------------------AIRKENAWAFEDLK-------- 170
ASK17 116 ADKTVNEIRELFNIENDYTPEEEE------------------------ELRKKNEWAFN----------- 150
ASK18 148 KDKTPEEVREIFNIENDFTPEEEE------------------------EIRKENAWTFNE---------- 183
ASK19 156 KDMTPEEVRELFNIENDFTPEEEE------------------------AIRNENAWTFEQDGKQQVPKP- 200
ASK20 132 EGKNPEEIREIFHLPDDLTEEEKLEPLKNSMDDPRIRLLNRLYAKKRKELKERERLKNVEVEEHVDERSV 201
ASK21 132 EGKTPEEIREIFHLPDDLTEEEKLEPLKNTMDDPRIRLLNRLYAKKRKELKEREKLKSVEVEEHVDERSV 201
OSK1 139 KGKTPEEIRKTFNIKNDFTPEEEE------------------------EIRRENQWAFE----------- 173
 . ..... . ...... ..... . .. ....

## Slide 3
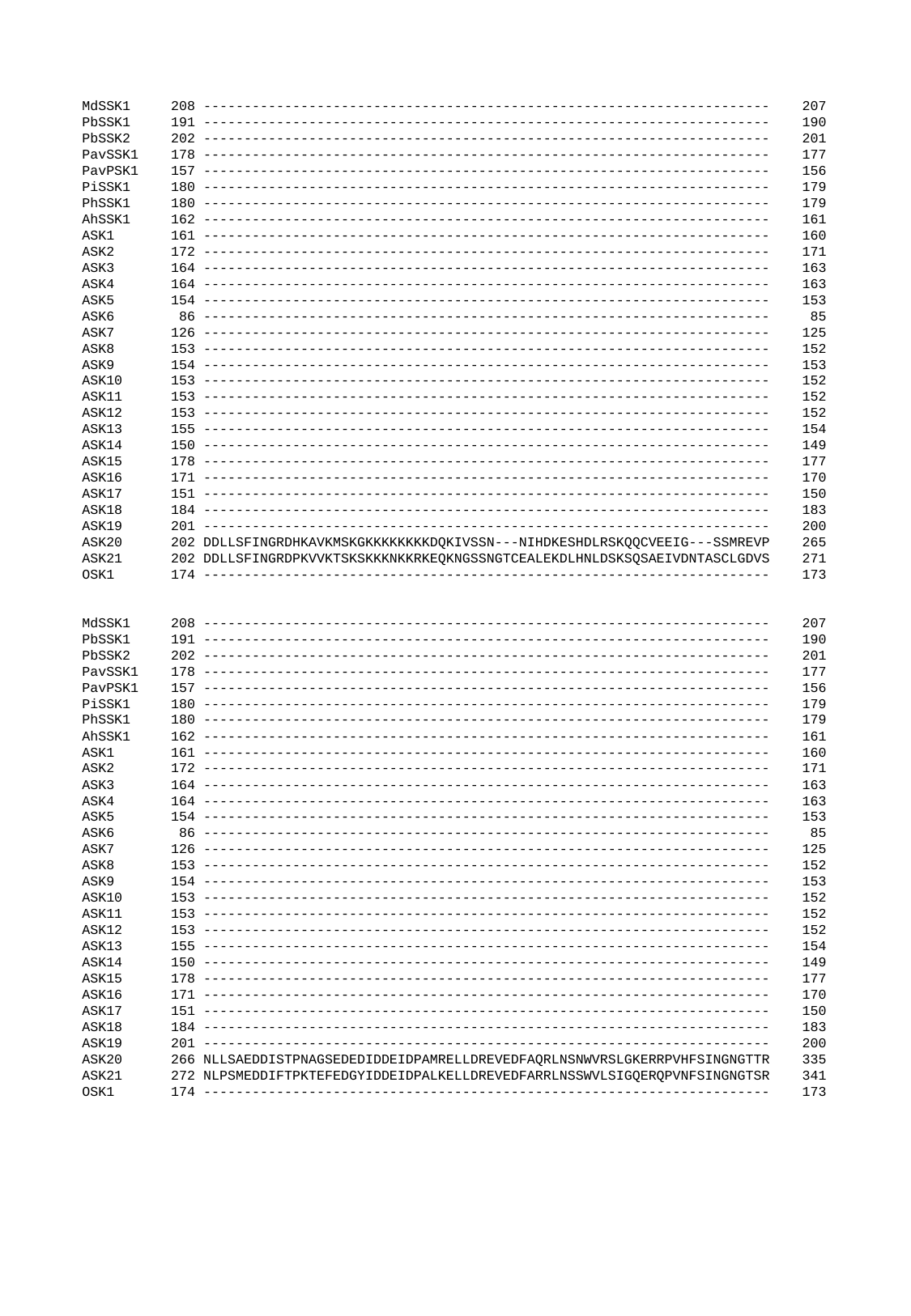

MdSSK1 208 ---------------------------------------------------------------------- 207
PbSSK1 191 ---------------------------------------------------------------------- 190
PbSSK2 202 ---------------------------------------------------------------------- 201
PavSSK1 178 ---------------------------------------------------------------------- 177
PavPSK1 157 ---------------------------------------------------------------------- 156
PiSSK1 180 ---------------------------------------------------------------------- 179
PhSSK1 180 ---------------------------------------------------------------------- 179
AhSSK1 162 ---------------------------------------------------------------------- 161
ASK1 161 ---------------------------------------------------------------------- 160
ASK2 172 ---------------------------------------------------------------------- 171
ASK3 164 ---------------------------------------------------------------------- 163
ASK4 164 ---------------------------------------------------------------------- 163
ASK5 154 ---------------------------------------------------------------------- 153
ASK6 86 ---------------------------------------------------------------------- 85
ASK7 126 ---------------------------------------------------------------------- 125
ASK8 153 ---------------------------------------------------------------------- 152
ASK9 154 ---------------------------------------------------------------------- 153
ASK10 153 ---------------------------------------------------------------------- 152
ASK11 153 ---------------------------------------------------------------------- 152
ASK12 153 ---------------------------------------------------------------------- 152
ASK13 155 ---------------------------------------------------------------------- 154
ASK14 150 ---------------------------------------------------------------------- 149
ASK15 178 ---------------------------------------------------------------------- 177
ASK16 171 ---------------------------------------------------------------------- 170
ASK17 151 ---------------------------------------------------------------------- 150
ASK18 184 ---------------------------------------------------------------------- 183
ASK19 201 ---------------------------------------------------------------------- 200
ASK20 202 DDLLSFINGRDHKAVKMSKGKKKKKKKKDQKIVSSN---NIHDKESHDLRSKQQCVEEIG---SSMREVP 265
ASK21 202 DDLLSFINGRDPKVVKTSKSKKKNKKRKEQKNGSSNGTCEALEKDLHNLDSKSQSAEIVDNTASCLGDVS 271
OSK1 174 ---------------------------------------------------------------------- 173
MdSSK1 208 ---------------------------------------------------------------------- 207
PbSSK1 191 ---------------------------------------------------------------------- 190
PbSSK2 202 ---------------------------------------------------------------------- 201
PavSSK1 178 ---------------------------------------------------------------------- 177
PavPSK1 157 ---------------------------------------------------------------------- 156
PiSSK1 180 ---------------------------------------------------------------------- 179
PhSSK1 180 ---------------------------------------------------------------------- 179
AhSSK1 162 ---------------------------------------------------------------------- 161
ASK1 161 ---------------------------------------------------------------------- 160
ASK2 172 ---------------------------------------------------------------------- 171
ASK3 164 ---------------------------------------------------------------------- 163
ASK4 164 ---------------------------------------------------------------------- 163
ASK5 154 ---------------------------------------------------------------------- 153
ASK6 86 ---------------------------------------------------------------------- 85
ASK7 126 ---------------------------------------------------------------------- 125
ASK8 153 ---------------------------------------------------------------------- 152
ASK9 154 ---------------------------------------------------------------------- 153
ASK10 153 ---------------------------------------------------------------------- 152
ASK11 153 ---------------------------------------------------------------------- 152
ASK12 153 ---------------------------------------------------------------------- 152
ASK13 155 ---------------------------------------------------------------------- 154
ASK14 150 ---------------------------------------------------------------------- 149
ASK15 178 ---------------------------------------------------------------------- 177
ASK16 171 ---------------------------------------------------------------------- 170
ASK17 151 ---------------------------------------------------------------------- 150
ASK18 184 ---------------------------------------------------------------------- 183
ASK19 201 ---------------------------------------------------------------------- 200
ASK20 266 NLLSAEDDISTPNAGSEDEDIDDEIDPAMRELLDREVEDFAQRLNSNWVRSLGKERRPVHFSINGNGTTR 335
ASK21 272 NLPSMEDDIFTPKTEFEDGYIDDEIDPALKELLDREVEDFARRLNSSWVLSIGQERQPVNFSINGNGTSR 341
OSK1 174 ---------------------------------------------------------------------- 173

## Slide 4
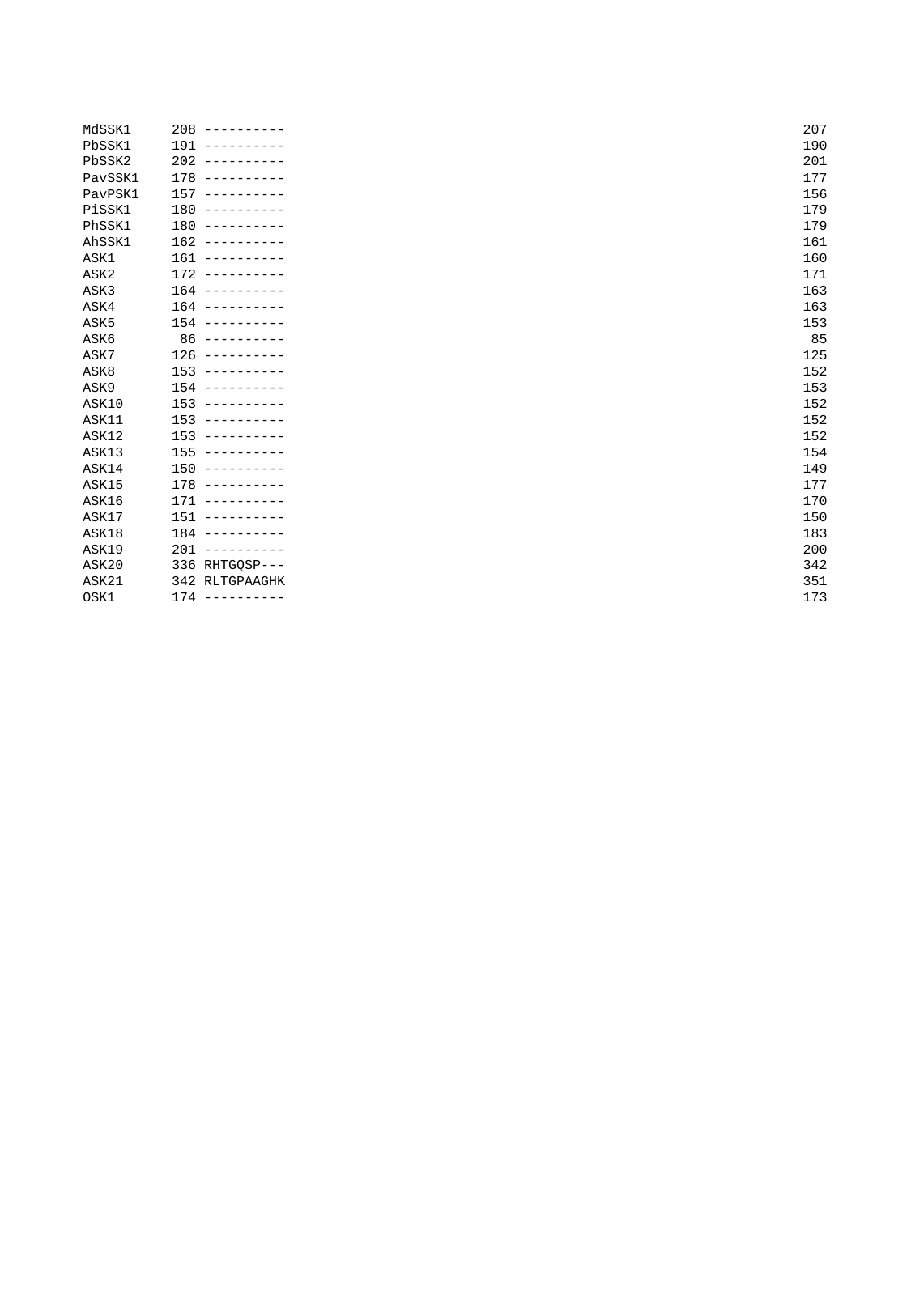

MdSSK1 208 ---------- 207
PbSSK1 191 ---------- 190
PbSSK2 202 ---------- 201
PavSSK1 178 ---------- 177
PavPSK1 157 ---------- 156
PiSSK1 180 ---------- 179
PhSSK1 180 ---------- 179
AhSSK1 162 ---------- 161
ASK1 161 ---------- 160
ASK2 172 ---------- 171
ASK3 164 ---------- 163
ASK4 164 ---------- 163
ASK5 154 ---------- 153
ASK6 86 ---------- 85
ASK7 126 ---------- 125
ASK8 153 ---------- 152
ASK9 154 ---------- 153
ASK10 153 ---------- 152
ASK11 153 ---------- 152
ASK12 153 ---------- 152
ASK13 155 ---------- 154
ASK14 150 ---------- 149
ASK15 178 ---------- 177
ASK16 171 ---------- 170
ASK17 151 ---------- 150
ASK18 184 ---------- 183
ASK19 201 ---------- 200
ASK20 336 RHTGQSP--- 342
ASK21 342 RLTGPAAGHK 351
OSK1 174 ---------- 173
