# Supplementary figures and images for: Identification of SFBB-Containing Canonical and Noncanonical SCF Complexes in Pollen of Apple (Malus × domestica)
Source: PLoS One. 2014 May 21;9(5):e97642. doi: 10.1371/journal.pone.0097642 (PMC4029751; doi:10.1371/journal.pone.0097642)

## Slide 1
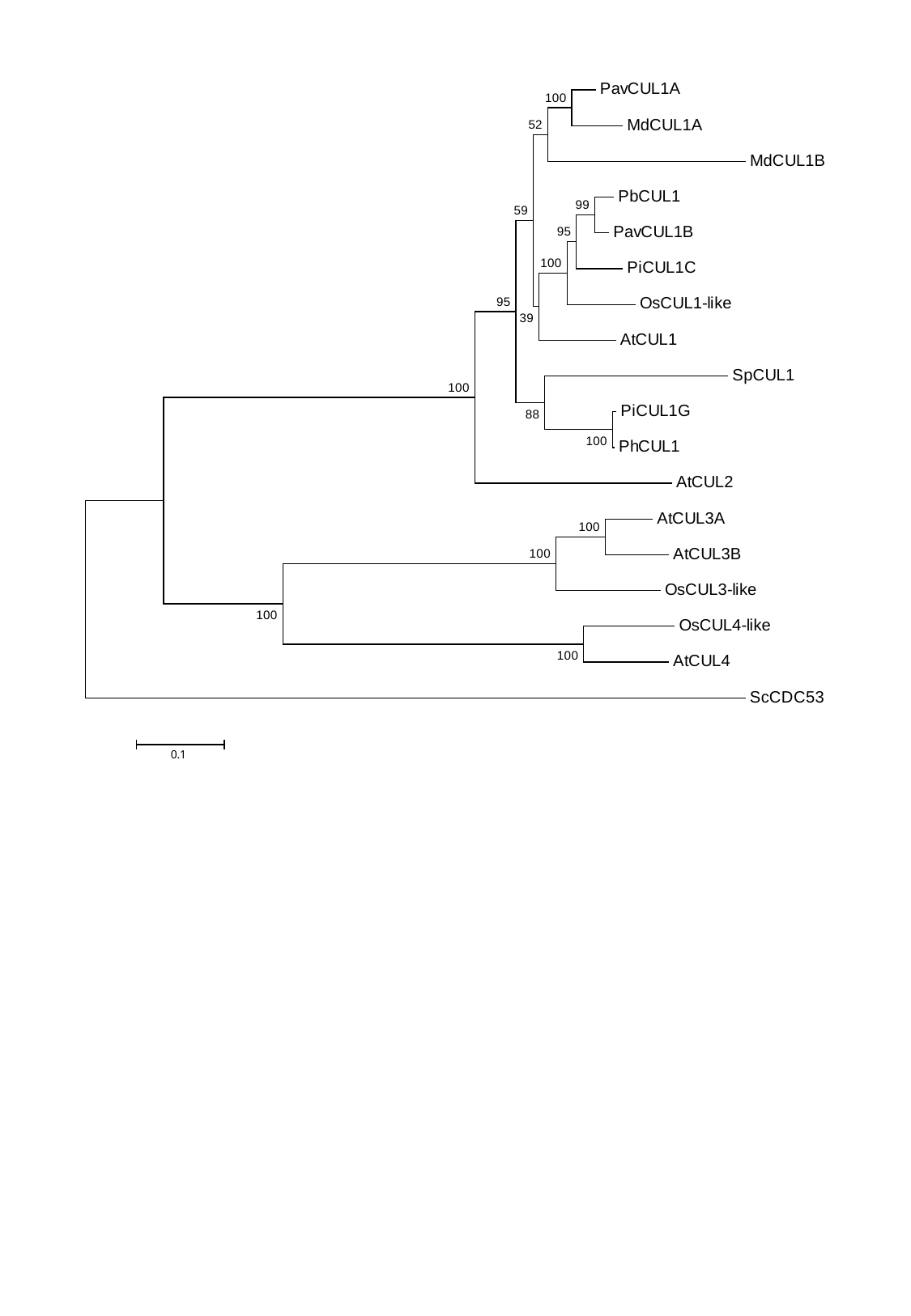

Supplement: Figure S2 — Neighbor-joining tree of MdCUL1s and other plant CUL-like proteins. The tree was constructed based on the aligned deduced amino acid sequences from apple (MdCUL1A, AB898684; MdCUL1B, AB898685), Pyrus bretschneideri (PbCUL1, CCH26221), sweet cherry (PavCUL1A, AFJ21664; PavCUL1B, AFJ21665), Petunia (PiCUL1G, ABB77429; PiCUL1C, ABB77428; PhCUL1, ACT35735), Solanum pennellii (SpCUL1, ADU60534), Arabidopsis (AtCUL1, NP_001031575; AtCUL2, NP_171797; AtCUL3A, NP_174005; AtCUL3B, NP_177125; AtCUL4, NP_568658) and rice (OsCUL1-like, LOC_Os01g27150; OsCUL3-like, LOC_Os02g51180; OsCUL4-like, LOC_Os03g57290). The tree was generated with 1000 bootstrap replicates. ScCDC53 (NP_010150) was defined as the outgroup. (PPTX) [file pone.0097642.s002.pptx]
