# Supplementary material for: Identification of SFBB-Containing Canonical and Noncanonical SCF Complexes in Pollen of Apple (Malus × domestica)
Source: PLoS One. 2014 May 21;9(5):e97642. doi: 10.1371/journal.pone.0097642 (PMC4029751; doi:10.1371/journal.pone.0097642)
Supplement: Figure S3 — Amino acid sequence alignment of MdCUL1 and other plant CUL-like proteins. Amino acid sequences were aligned using Clustal W. Conserved sites and relatively conservative sites are marked with asterisks and dots, respectively. The NEDD8 domain detected by Pfam (http://pfam.sanger.ac.uk) is denoted by a line. Abbreviations: Md, Malus × domestica; Pb, Pyrus bretschneideri; Pav, Prunus avium; Pi, Petunia inflata; Ph, Petunia hybrida; Sp, Solanum pennellii; At, Arabidopsis thaliana; Os, Oryza sativa and Sc, Saccharomyces cerevisiae. Accession numbers: PbCUL1 (CCH26221), PavCUL1A (AFJ21664), PavCUL1B (AFJ21665), PiCUL1G (ABB77429), PiCUL1C (ABB77428), PhCUL1 (ACT35735), SpCUL1 (ADU60534), AtCUL1 (NP_001031575), AtCUL2 (NP_171797), AtCUL3A (NP_174005), AtCUL3B (NP_177125), AtCUL4 (NP_568658), OsCUL1-like (LOC_Os01g27150), OsCUL3-like (LOC_Os02g51180), OsCUL4-like (LOC_Os03g57290), ScCDC53 (NP_010150). (PPTX) [file pone.0097642.s003.pptx]

## Slide 1
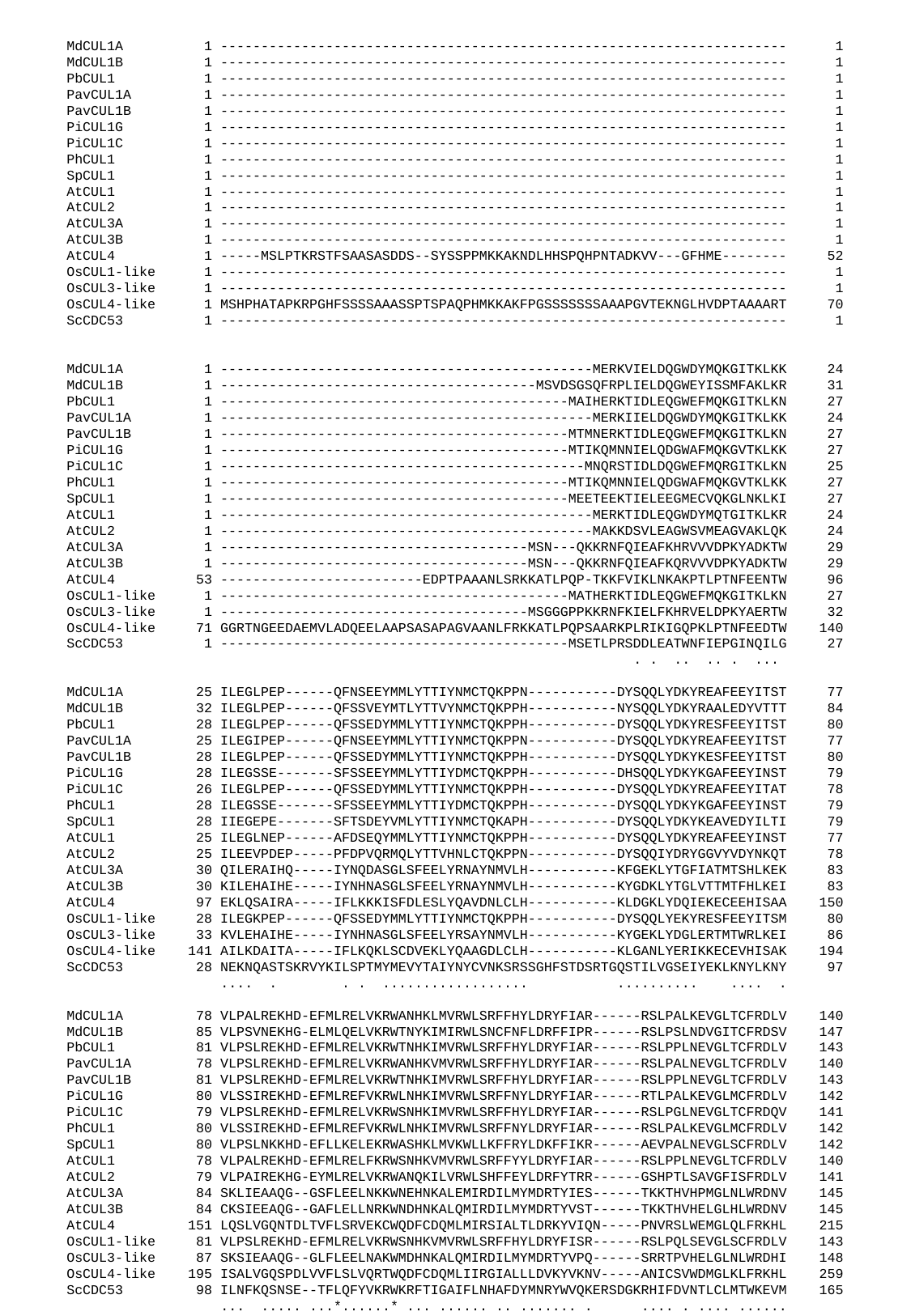

MdCUL1A 1 ---------------------------------------------------------------------- 1
MdCUL1B 1 ---------------------------------------------------------------------- 1
PbCUL1 1 ---------------------------------------------------------------------- 1
PavCUL1A 1 ---------------------------------------------------------------------- 1
PavCUL1B 1 ---------------------------------------------------------------------- 1
PiCUL1G 1 ---------------------------------------------------------------------- 1
PiCUL1C 1 ---------------------------------------------------------------------- 1
PhCUL1 1 ---------------------------------------------------------------------- 1
SpCUL1 1 ---------------------------------------------------------------------- 1
AtCUL1 1 ---------------------------------------------------------------------- 1
AtCUL2 1 ---------------------------------------------------------------------- 1
AtCUL3A 1 ---------------------------------------------------------------------- 1
AtCUL3B 1 ---------------------------------------------------------------------- 1
AtCUL4 1 -----MSLPTKRSTFSAASASDDS--SYSSPPMKKAKNDLHHSPQHPNTADKVV---GFHME-------- 52
OsCUL1-like 1 ---------------------------------------------------------------------- 1
OsCUL3-like 1 ---------------------------------------------------------------------- 1
OsCUL4-like 1 MSHPHATAPKRPGHFSSSSAAASSPTSPAQPHMKKAKFPGSSSSSSSAAAPGVTEKNGLHVDPTAAAART 70
ScCDC53 1 ---------------------------------------------------------------------- 1
MdCUL1A 1 ----------------------------------------------MERKVIELDQGWDYMQKGITKLKK 24
MdCUL1B 1 ---------------------------------------MSVDSGSQFRPLIELDQGWEYISSMFAKLKR 31
PbCUL1 1 -------------------------------------------MAIHERKTIDLEQGWEFMQKGITKLKN 27
PavCUL1A 1 ----------------------------------------------MERKIIELDQGWDYMQKGITKLKK 24
PavCUL1B 1 -------------------------------------------MTMNERKTIDLEQGWEFMQKGITKLKN 27
PiCUL1G 1 -------------------------------------------MTIKQMNNIELQDGWAFMQKGVTKLKK 27
PiCUL1C 1 ---------------------------------------------MNQRSTIDLDQGWEFMQRGITKLKN 25
PhCUL1 1 -------------------------------------------MTIKQMNNIELQDGWAFMQKGVTKLKK 27
SpCUL1 1 -------------------------------------------MEETEEKTIELEEGMECVQKGLNKLKI 27
AtCUL1 1 ----------------------------------------------MERKTIDLEQGWDYMQTGITKLKR 24
AtCUL2 1 ----------------------------------------------MAKKDSVLEAGWSVMEAGVAKLQK 24
AtCUL3A 1 --------------------------------------MSN---QKKRNFQIEAFKHRVVVDPKYADKTW 29
AtCUL3B 1 --------------------------------------MSN---QKKRNFQIEAFKQRVVVDPKYADKTW 29
AtCUL4 53 -------------------------EDPTPAAANLSRKKATLPQP-TKKFVIKLNKAKPTLPTNFEENTW 96
OsCUL1-like 1 -------------------------------------------MATHERKTIDLEQGWEFMQKGITKLKN 27
OsCUL3-like 1 --------------------------------------MSGGGPPKKRNFKIELFKHRVELDPKYAERTW 32
OsCUL4-like 71 GGRTNGEEDAEMVLADQEELAAPSASAPAGVAANLFRKKATLPQPSAARKPLRIKIGQPKLPTNFEEDTW 140
ScCDC53 1 -------------------------------------------MSETLPRSDDLEATWNFIEPGINQILG 27
 . . .. .. . ...
MdCUL1A 25 ILEGLPEP------QFNSEEYMMLYTTIYNMCTQKPPN-----------DYSQQLYDKYREAFEEYITST 77
MdCUL1B 32 ILEGLPEP------QFSSVEYMTLYTTVYNMCTQKPPH-----------NYSQQLYDKYRAALEDYVTTT 84
PbCUL1 28 ILEGLPEP------QFSSEDYMMLYTTIYNMCTQKPPH-----------DYSQQLYDKYRESFEEYITST 80
PavCUL1A 25 ILEGIPEP------QFNSEEYMMLYTTIYNMCTQKPPN-----------DYSQQLYDKYREAFEEYITST 77
PavCUL1B 28 ILEGLPEP------QFSSEDYMMLYTTIYNMCTQKPPH-----------DYSQQLYDKYKESFEEYITST 80
PiCUL1G 28 ILEGSSE-------SFSSEEYMMLYTTIYDMCTQKPPH-----------DHSQQLYDKYKGAFEEYINST 79
PiCUL1C 26 ILEGLPEP------QFSSEDYMMLYTTIYNMCTQKPPH-----------DYSQQLYDKYREAFEEYITAT 78
PhCUL1 28 ILEGSSE-------SFSSEEYMMLYTTIYDMCTQKPPH-----------DYSQQLYDKYKGAFEEYINST 79
SpCUL1 28 IIEGEPE-------SFTSDEYVMLYTTIYNMCTQKAPH-----------DYSQQLYDKYKEAVEDYILTI 79
AtCUL1 25 ILEGLNEP------AFDSEQYMMLYTTIYNMCTQKPPH-----------DYSQQLYDKYREAFEEYINST 77
AtCUL2 25 ILEEVPDEP-----PFDPVQRMQLYTTVHNLCTQKPPN-----------DYSQQIYDRYGGVYVDYNKQT 78
AtCUL3A 30 QILERAIHQ-----IYNQDASGLSFEELYRNAYNMVLH-----------KFGEKLYTGFIATMTSHLKEK 83
AtCUL3B 30 KILEHAIHE-----IYNHNASGLSFEELYRNAYNMVLH-----------KYGDKLYTGLVTTMTFHLKEI 83
AtCUL4 97 EKLQSAIRA-----IFLKKKISFDLESLYQAVDNLCLH-----------KLDGKLYDQIEKECEEHISAA 150
OsCUL1-like 28 ILEGKPEP------QFSSEDYMMLYTTIYNMCTQKPPH-----------DYSQQLYEKYRESFEEYITSM 80
OsCUL3-like 33 KVLEHAIHE-----IYNHNASGLSFEELYRSAYNMVLH-----------KYGEKLYDGLERTMTWRLKEI 86
OsCUL4-like 141 AILKDAITA-----IFLKQKLSCDVEKLYQAAGDLCLH-----------KLGANLYERIKKECEVHISAK 194
ScCDC53 28 NEKNQASTSKRVYKILSPTMYMEVYTAIYNYCVNKSRSSGHFSTDSRTGQSTILVGSEIYEKLKNYLKNY 97
 .... . . . .................. .......... .... .
MdCUL1A 78 VLPALREKHD-EFMLRELVKRWANHKLMVRWLSRFFHYLDRYFIAR------RSLPALKEVGLTCFRDLV 140
MdCUL1B 85 VLPSVNEKHG-ELMLQELVKRWTNYKIMIRWLSNCFNFLDRFFIPR------RSLPSLNDVGITCFRDSV 147
PbCUL1 81 VLPSLREKHD-EFMLRELVKRWTNHKIMVRWLSRFFHYLDRYFIAR------RSLPPLNEVGLTCFRDLV 143
PavCUL1A 78 VLPSLREKHD-EFMLRELVKRWANHKVMVRWLSRFFHYLDRYFIAR------RSLPALNEVGLTCFRDLV 140
PavCUL1B 81 VLPSLREKHD-EFMLRELVKRWTNHKIMVRWLSRFFHYLDRYFIAR------RSLPPLNEVGLTCFRDLV 143
PiCUL1G 80 VLSSIREKHD-EFMLREFVKRWLNHKIMVRWLSRFFNYLDRYFIAR------RTLPALKEVGLMCFRDLV 142
PiCUL1C 79 VLPSLREKHD-EFMLRELVKRWSNHKIMVRWLSRFFHYLDRYFIAR------RSLPGLNEVGLTCFRDQV 141
PhCUL1 80 VLSSIREKHD-EFMLREFVKRWLNHKIMVRWLSRFFNYLDRYFIAR------RSLPALKEVGLMCFRDLV 142
SpCUL1 80 VLPSLNKKHD-EFLLKELEKRWASHKLMVKWLLKFFRYLDKFFIKR------AEVPALNEVGLSCFRDLV 142
AtCUL1 78 VLPALREKHD-EFMLRELFKRWSNHKVMVRWLSRFFYYLDRYFIAR------RSLPPLNEVGLTCFRDLV 140
AtCUL2 79 VLPAIREKHG-EYMLRELVKRWANQKILVRWLSHFFEYLDRFYTRR------GSHPTLSAVGFISFRDLV 141
AtCUL3A 84 SKLIEAAQG--GSFLEELNKKWNEHNKALEMIRDILMYMDRTYIES------TKKTHVHPMGLNLWRDNV 145
AtCUL3B 84 CKSIEEAQG--GAFLELLNRKWNDHNKALQMIRDILMYMDRTYVST------TKKTHVHELGLHLWRDNV 145
AtCUL4 151 LQSLVGQNTDLTVFLSRVEKCWQDFCDQMLMIRSIALTLDRKYVIQN-----PNVRSLWEMGLQLFRKHL 215
OsCUL1-like 81 VLPSLREKHD-EFMLRELVKRWSNHKVMVRWLSRFFHYLDRYFISR------RSLPQLSEVGLSCFRDLV 143
OsCUL3-like 87 SKSIEAAQG--GLFLEELNAKWMDHNKALQMIRDILMYMDRTYVPQ------SRRTPVHELGLNLWRDHI 148
OsCUL4-like 195 ISALVGQSPDLVVFLSLVQRTWQDFCDQMLIIRGIALLLDVKYVKNV-----ANICSVWDMGLKLFRKHL 259
ScCDC53 98 ILNFKQSNSE--TFLQFYVKRWKRFTIGAIFLNHAFDYMNRYWVQKERSDGKRHIFDVNTLCLMTWKEVM 165
 ... ..... ...*......* ... ...... .. ....... . .... . .... ......

## Slide 2
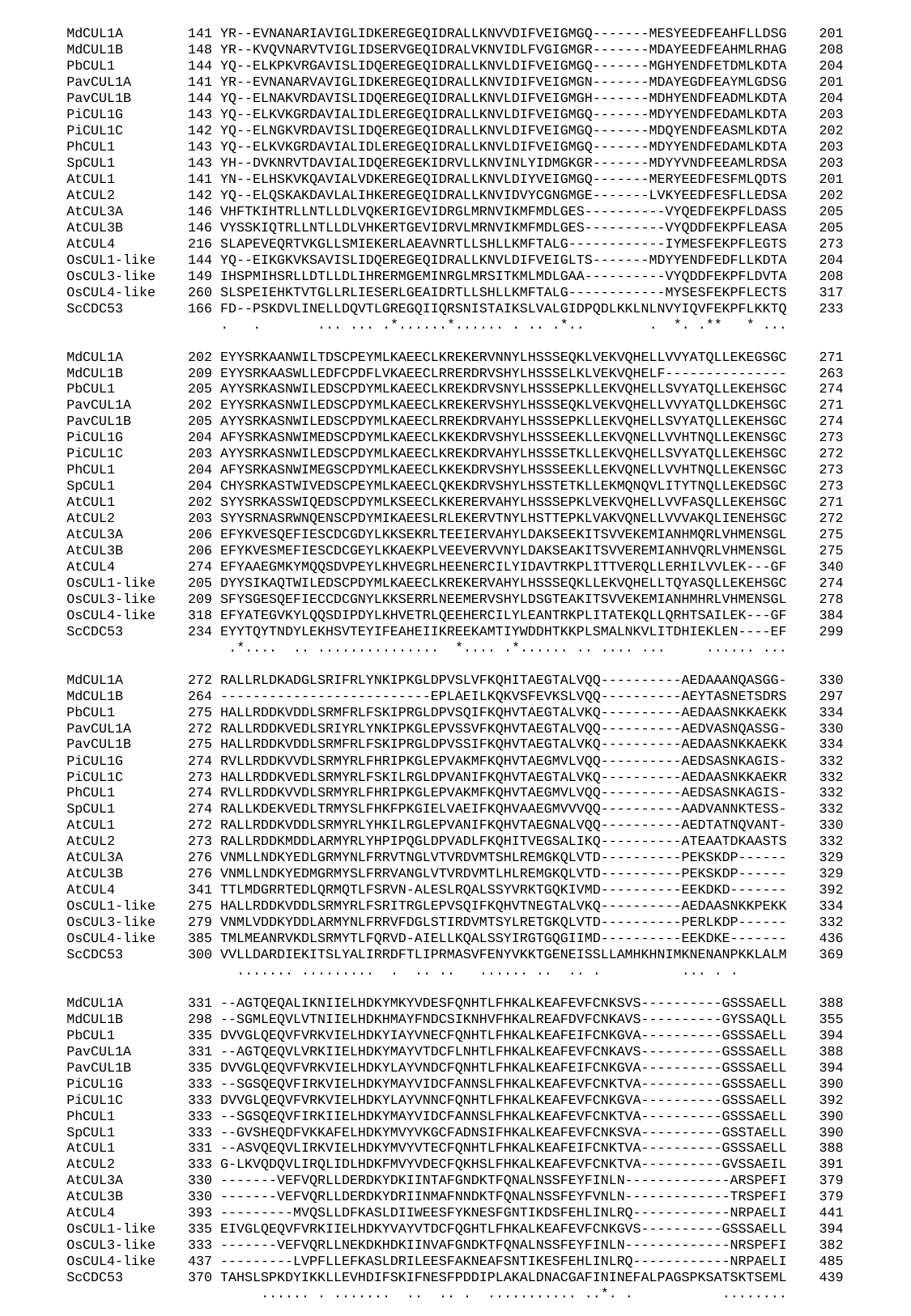

MdCUL1A 141 YR--EVNANARIAVIGLIDKEREGEQIDRALLKNVVDIFVEIGMGQ-------MESYEEDFEAHFLLDSG 201
MdCUL1B 148 YR--KVQVNARVTVIGLIDSERVGEQIDRALVKNVIDLFVGIGMGR-------MDAYEEDFEAHMLRHAG 208
PbCUL1 144 YQ--ELKPKVRGAVISLIDQEREGEQIDRALLKNVLDIFVEIGMGQ-------MGHYENDFETDMLKDTA 204
PavCUL1A 141 YR--EVNANARVAVIGLIDKEREGEQIDRALLKNVIDIFVEIGMGN-------MDAYEGDFEAYMLGDSG 201
PavCUL1B 144 YQ--ELNAKVRDAVISLIDQEREGEQIDRALLKNVLDIFVEIGMGH-------MDHYENDFEADMLKDTA 204
PiCUL1G 143 YQ--ELKVKGRDAVIALIDLEREGEQIDRALLKNVLDIFVEIGMGQ-------MDYYENDFEDAMLKDTA 203
PiCUL1C 142 YQ--ELNGKVRDAVISLIDQEREGEQIDRALLKNVLDIFVEIGMGQ-------MDQYENDFEASMLKDTA 202
PhCUL1 143 YQ--ELKVKGRDAVIALIDLEREGEQIDRALLKNVLDIFVEIGMGQ-------MDYYENDFEDAMLKDTA 203
SpCUL1 143 YH--DVKNRVTDAVIALIDQEREGEKIDRVLLKNVINLYIDMGKGR-------MDYYVNDFEEAMLRDSA 203
AtCUL1 141 YN--ELHSKVKQAVIALVDKEREGEQIDRALLKNVLDIYVEIGMGQ-------MERYEEDFESFMLQDTS 201
AtCUL2 142 YQ--ELQSKAKDAVLALIHKEREGEQIDRALLKNVIDVYCGNGMGE-------LVKYEEDFESFLLEDSA 202
AtCUL3A 146 VHFTKIHTRLLNTLLDLVQKERIGEVIDRGLMRNVIKMFMDLGES----------VYQEDFEKPFLDASS 205
AtCUL3B 146 VYSSKIQTRLLNTLLDLVHKERTGEVIDRVLMRNVIKMFMDLGES----------VYQDDFEKPFLEASA 205
AtCUL4 216 SLAPEVEQRTVKGLLSMIEKERLAEAVNRTLLSHLLKMFTALG------------IYMESFEKPFLEGTS 273
OsCUL1-like 144 YQ--EIKGKVKSAVISLIDQEREGEQIDRALLKNVLDIFVEIGLTS-------MDYYENDFEDFLLKDTA 204
OsCUL3-like 149 IHSPMIHSRLLDTLLDLIHRERMGEMINRGLMRSITKMLMDLGAA----------VYQDDFEKPFLDVTA 208
OsCUL4-like 260 SLSPEIEHKTVTGLLRLIESERLGEAIDRTLLSHLLKMFTALG------------MYSESFEKPFLECTS 317
ScCDC53 166 FD--PSKDVLINELLDQVTLGREGQIIQRSNISTAIKSLVALGIDPQDLKKLNLNVYIQVFEKPFLKKTQ 233
 . . ... ... .*......*...... . .. .*.. . *. .** * ...
MdCUL1A 202 EYYSRKAANWILTDSCPEYMLKAEECLKREKERVNNYLHSSSEQKLVEKVQHELLVVYATQLLEKEGSGC 271
MdCUL1B 209 EYYSRKAASWLLEDFCPDFLVKAEECLRRERDRVSHYLHSSSELKLVEKVQHELF--------------- 263
PbCUL1 205 AYYSRKASNWILEDSCPDYMLKAEECLKREKDRVSNYLHSSSEPKLLEKVQHELLSVYATQLLEKEHSGC 274
PavCUL1A 202 EYYSRKASNWILEDSCPDYMLKAEECLKREKERVSHYLHSSSEQKLVEKVQHELLVVYATQLLDKEHSGC 271
PavCUL1B 205 AYYSRKASNWILEDSCPDYMLKAEECLRREKDRVAHYLHSSSEPKLLEKVQHELLSVYATQLLEKEHSGC 274
PiCUL1G 204 AFYSRKASNWIMEDSCPDYMLKAEECLKKEKDRVSHYLHSSSEEKLLEKVQNELLVVHTNQLLEKENSGC 273
PiCUL1C 203 AYYSRKASNWILEDSCPDYMLKAEECLKREKDRVAHYLHSSSETKLLEKVQHELLSVYATQLLEKEHSGC 272
PhCUL1 204 AFYSRKASNWIMEGSCPDYMLKAEECLKKEKDRVSHYLHSSSEEKLLEKVQNELLVVHTNQLLEKENSGC 273
SpCUL1 204 CHYSRKASTWIVEDSCPEYMLKAEECLQKEKDRVSHYLHSSTETKLLEKMQNQVLITYTNQLLEKEDSGC 273
AtCUL1 202 SYYSRKASSWIQEDSCPDYMLKSEECLKKERERVAHYLHSSSEPKLVEKVQHELLVVFASQLLEKEHSGC 271
AtCUL2 203 SYYSRNASRWNQENSCPDYMIKAEESLRLEKERVTNYLHSTTEPKLVAKVQNELLVVVAKQLIENEHSGC 272
AtCUL3A 206 EFYKVESQEFIESCDCGDYLKKSEKRLTEEIERVAHYLDAKSEEKITSVVEKEMIANHMQRLVHMENSGL 275
AtCUL3B 206 EFYKVESMEFIESCDCGEYLKKAEKPLVEEVERVVNYLDAKSEAKITSVVEREMIANHVQRLVHMENSGL 275
AtCUL4 274 EFYAAEGMKYMQQSDVPEYLKHVEGRLHEENERCILYIDAVTRKPLITTVERQLLERHILVVLEK---GF 340
OsCUL1-like 205 DYYSIKAQTWILEDSCPDYMLKAEECLKREKERVAHYLHSSSEQKLLEKVQHELLTQYASQLLEKEHSGC 274
OsCUL3-like 209 SFYSGESQEFIECCDCGNYLKKSERRLNEEMERVSHYLDSGTEAKITSVVEKEMIANHMHRLVHMENSGL 278
OsCUL4-like 318 EFYATEGVKYLQQSDIPDYLKHVETRLQEEHERCILYLEANTRKPLITATEKQLLQRHTSAILEK---GF 384
ScCDC53 234 EYYTQYTNDYLEKHSVTEYIFEAHEIIKREEKAMTIYWDDHTKKPLSMALNKVLITDHIEKLEN----EF 299
 .*.... .. ............... *.... .*...... .. .... ... ...... ...
MdCUL1A 272 RALLRLDKADGLSRIFRLYNKIPKGLDPVSLVFKQHITAEGTALVQQ----------AEDAAANQASGG- 330
MdCUL1B 264 --------------------------EPLAEILKQKVSFEVKSLVQQ----------AEYTASNETSDRS 297
PbCUL1 275 HALLRDDKVDDLSRMFRLFSKIPRGLDPVSQIFKQHVTAEGTALVKQ----------AEDAASNKKAEKK 334
PavCUL1A 272 RALLRDDKVEDLSRIYRLYNKIPKGLEPVSSVFKQHVTAEGTALVQQ----------AEDVASNQASSG- 330
PavCUL1B 275 HALLRDDKVDDLSRMFRLFSKIPRGLDPVSSIFKQHVTAEGTALVKQ----------AEDAASNKKAEKK 334
PiCUL1G 274 RVLLRDDKVVDLSRMYRLFHRIPKGLEPVAKMFKQHVTAEGMVLVQQ----------AEDSASNKAGIS- 332
PiCUL1C 273 HALLRDDKVEDLSRMYRLFSKILRGLDPVANIFKQHVTAEGTALVKQ----------AEDAASNKKAEKR 332
PhCUL1 274 RVLLRDDKVVDLSRMYRLFHRIPKGLEPVAKMFKQHVTAEGMVLVQQ----------AEDSASNKAGIS- 332
SpCUL1 274 RALLKDEKVEDLTRMYSLFHKFPKGIELVAEIFKQHVAAEGMVVVQQ----------AADVANNKTESS- 332
AtCUL1 272 RALLRDDKVDDLSRMYRLYHKILRGLEPVANIFKQHVTAEGNALVQQ----------AEDTATNQVANT- 330
AtCUL2 273 RALLRDDKMDDLARMYRLYHPIPQGLDPVADLFKQHITVEGSALIKQ----------ATEAATDKAASTS 332
AtCUL3A 276 VNMLLNDKYEDLGRMYNLFRRVTNGLVTVRDVMTSHLREMGKQLVTD----------PEKSKDP------ 329
AtCUL3B 276 VNMLLNDKYEDMGRMYSLFRRVANGLVTVRDVMTLHLREMGKQLVTD----------PEKSKDP------ 329
AtCUL4 341 TTLMDGRRTEDLQRMQTLFSRVN-ALESLRQALSSYVRKTGQKIVMD----------EEKDKD------- 392
OsCUL1-like 275 HALLRDDKVDDLSRMYRLFSRITRGLEPVSQIFKQHVTNEGTALVKQ----------AEDAASNKKPEKK 334
OsCUL3-like 279 VNMLVDDKYDDLARMYNLFRRVFDGLSTIRDVMTSYLRETGKQLVTD----------PERLKDP------ 332
OsCUL4-like 385 TMLMEANRVKDLSRMYTLFQRVD-AIELLKQALSSYIRGTGQGIIMD----------EEKDKE------- 436
ScCDC53 300 VVLLDARDIEKITSLYALIRRDFTLIPRMASVFENYVKKTGENEISSLLAMHKHNIMKNENANPKKLALM 369
 ....... ......... . .. .. ...... .. .. . ... . .
MdCUL1A 331 --AGTQEQALIKNIIELHDKYMKYVDESFQNHTLFHKALKEAFEVFCNKSVS----------GSSSAELL 388
MdCUL1B 298 --SGMLEQVLVTNIIELHDKHMAYFNDCSIKNHVFHKALREAFDVFCNKAVS----------GYSSAQLL 355
PbCUL1 335 DVVGLQEQVFVRKVIELHDKYIAYVNECFQNHTLFHKALKEAFEIFCNKGVA----------GSSSAELL 394
PavCUL1A 331 --AGTQEQVLVRKIIELHDKYMAYVTDCFLNHTLFHKALKEAFEVFCNKAVS----------GSSSAELL 388
PavCUL1B 335 DVVGLQEQVFVRKVIELHDKYLAYVNDCFQNHTLFHKALKEAFEIFCNKGVA----------GSSSAELL 394
PiCUL1G 333 --SGSQEQVFIRKVIELHDKYMAYVIDCFANNSLFHKALKEAFEVFCNKTVA----------GSSSAELL 390
PiCUL1C 333 DVVGLQEQVFVRKVIELHDKYLAYVNNCFQNHTLFHKALKEAFEVFCNKGVA----------GSSSAELL 392
PhCUL1 333 --SGSQEQVFIRKIIELHDKYMAYVIDCFANNSLFHKALKEAFEVFCNKTVA----------GSSSAELL 390
SpCUL1 333 --GVSHEQDFVKKAFELHDKYMVYVKGCFADNSIFHKALKEAFEVFCNKSVA----------GSSTAELL 390
AtCUL1 331 --ASVQEQVLIRKVIELHDKYMVYVTECFQNHTLFHKALKEAFEIFCNKTVA----------GSSSAELL 388
AtCUL2 333 G-LKVQDQVLIRQLIDLHDKFMVYVDECFQKHSLFHKALKEAFEVFCNKTVA----------GVSSAEIL 391
AtCUL3A 330 -------VEFVQRLLDERDKYDKIINTAFGNDKTFQNALNSSFEYFINLN-------------ARSPEFI 379
AtCUL3B 330 -------VEFVQRLLDERDKYDRIINMAFNNDKTFQNALNSSFEYFVNLN-------------TRSPEFI 379
AtCUL4 393 ---------MVQSLLDFKASLDIIWEESFYKNESFGNTIKDSFEHLINLRQ------------NRPAELI 441
OsCUL1-like 335 EIVGLQEQVFVRKIIELHDKYVAYVTDCFQGHTLFHKALKEAFEVFCNKGVS----------GSSSAELL 394
OsCUL3-like 333 -------VEFVQRLLNEKDKHDKIINVAFGNDKTFQNALNSSFEYFINLN-------------NRSPEFI 382
OsCUL4-like 437 ---------LVPFLLEFKASLDRILEESFAKNEAFSNTIKESFEHLINLRQ------------NRPAELI 485
ScCDC53 370 TAHSLSPKDYIKKLLEVHDIFSKIFNESFPDDIPLAKALDNACGAFININEFALPAGSPKSATSKTSEML 439
 ...... . ....... .. .. . ........... ..*. . ........

## Slide 3
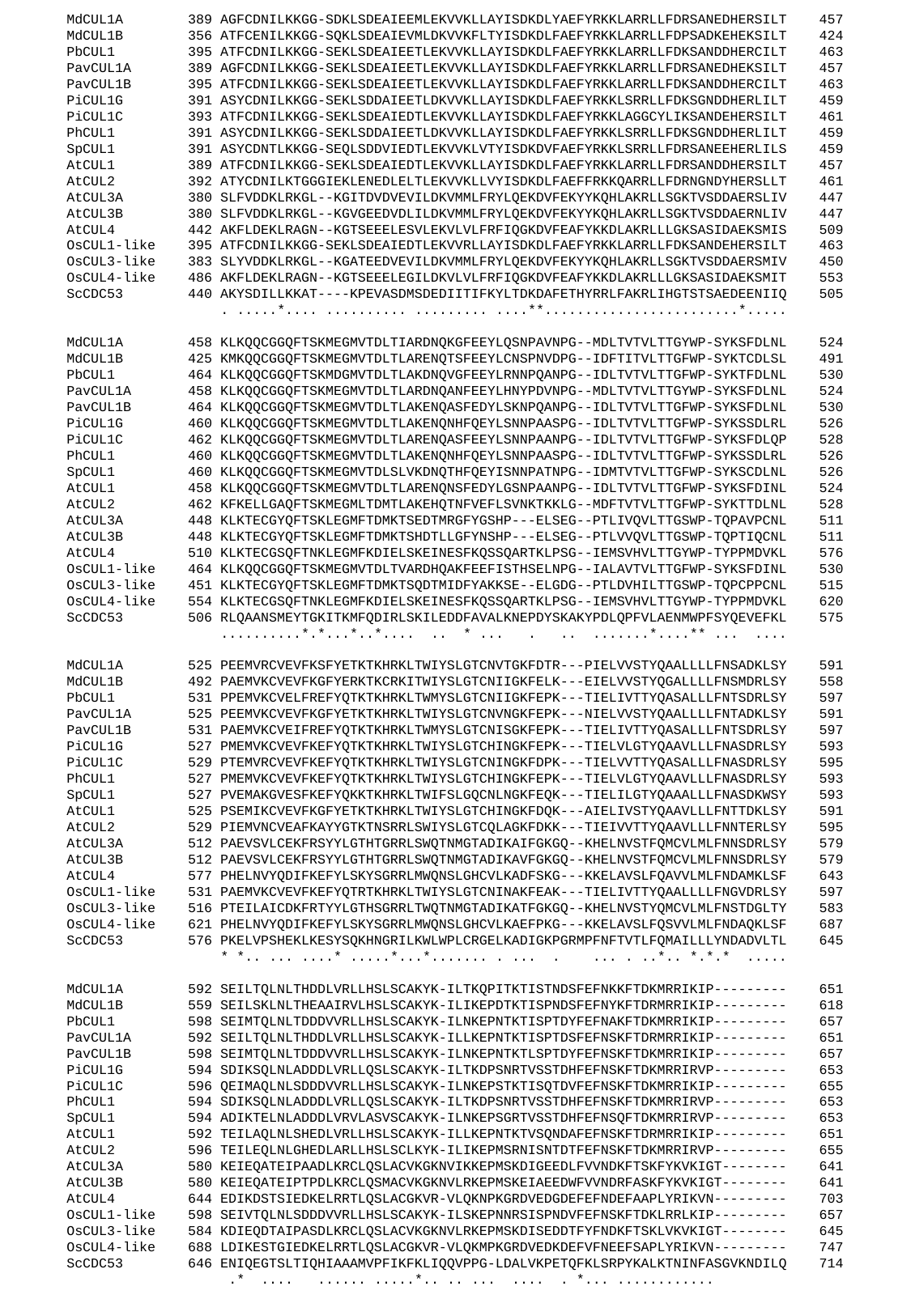

MdCUL1A 389 AGFCDNILKKGG-SDKLSDEAIEEMLEKVVKLLAYISDKDLYAEFYRKKLARRLLFDRSANEDHERSILT 457
MdCUL1B 356 ATFCENILKKGG-SQKLSDEAIEVMLDKVVKFLTYISDKDLFAEFYRKKLARRLLFDPSADKEHEKSILT 424
PbCUL1 395 ATFCDNILKKGG-SEKLSDEAIEETLEKVVKLLAYISDKDLFAEFYRKKLARRLLFDKSANDDHERCILT 463
PavCUL1A 389 AGFCDNILKKGG-SEKLSDEAIEETLEKVVKLLAYISDKDLFAEFYRKKLARRLLFDRSANEDHEKSILT 457
PavCUL1B 395 ATFCDNILKKGG-SEKLSDEAIEETLEKVVKLLAYISDKDLFAEFYRKKLARRLLFDKSANDDHERCILT 463
PiCUL1G 391 ASYCDNILKKGG-SEKLSDDAIEETLDKVVKLLAYISDKDLFAEFYRKKLSRRLLFDKSGNDDHERLILT 459
PiCUL1C 393 ATFCDNILKKGG-SEKLSDEAIEDTLEKVVKLLAYISDKDLFAEFYRKKLAGGCYLIKSANDEHERSILT 461
PhCUL1 391 ASYCDNILKKGG-SEKLSDDAIEETLDKVVKLLAYISDKDLFAEFYRKKLSRRLLFDKSGNDDHERLILT 459
SpCUL1 391 ASYCDNTLKKGG-SEQLSDDVIEDTLEKVVKLVTYISDKDVFAEFYRKKLSRRLLFDRSANEEHERLILS 459
AtCUL1 389 ATFCDNILKKGG-SEKLSDEAIEDTLEKVVKLLAYISDKDLFAEFYRKKLARRLLFDRSANDDHERSILT 457
AtCUL2 392 ATYCDNILKTGGGIEKLENEDLELTLEKVVKLLVYISDKDLFAEFFRKKQARRLLFDRNGNDYHERSLLT 461
AtCUL3A 380 SLFVDDKLRKGL--KGITDVDVEVILDKVMMLFRYLQEKDVFEKYYKQHLAKRLLSGKTVSDDAERSLIV 447
AtCUL3B 380 SLFVDDKLRKGL--KGVGEEDVDLILDKVMMLFRYLQEKDVFEKYYKQHLAKRLLSGKTVSDDAERNLIV 447
AtCUL4 442 AKFLDEKLRAGN--KGTSEEELESVLEKVLVLFRFIQGKDVFEAFYKKDLAKRLLLGKSASIDAEKSMIS 509
OsCUL1-like 395 ATFCDNILKKGG-SEKLSDEAIEDTLEKVVRLLAYISDKDLFAEFYRKKLARRLLFDKSANDEHERSILT 463
OsCUL3-like 383 SLYVDDKLRKGL--KGATEEDVEVILDKVMMLFRYLQEKDVFEKYYKQHLAKRLLSGKTVSDDAERSMIV 450
OsCUL4-like 486 AKFLDEKLRAGN--KGTSEEELEGILDKVLVLFRFIQGKDVFEAFYKKDLAKRLLLGKSASIDAEKSMIT 553
ScCDC53 440 AKYSDILLKKAT----KPEVASDMSDEDIITIFKYLTDKDAFETHYRRLFAKRLIHGTSTSAEDEENIIQ 505
 . .....*.... .......... ......... ....**........................*.....
MdCUL1A 458 KLKQQCGGQFTSKMEGMVTDLTIARDNQKGFEEYLQSNPAVNPG--MDLTVTVLTTGYWP-SYKSFDLNL 524
MdCUL1B 425 KMKQQCGGQFTSKMEGMVTDLTLARENQTSFEEYLCNSPNVDPG--IDFTITVLTTGFWP-SYKTCDLSL 491
PbCUL1 464 KLKQQCGGQFTSKMDGMVTDLTLAKDNQVGFEEYLRNNPQANPG--IDLTVTVLTTGFWP-SYKTFDLNL 530
PavCUL1A 458 KLKQQCGGQFTSKMEGMVTDLTLARDNQANFEEYLHNYPDVNPG--MDLTVTVLTTGYWP-SYKSFDLNL 524
PavCUL1B 464 KLKQQCGGQFTSKMEGMVTDLTLAKENQASFEDYLSKNPQANPG--IDLTVTVLTTGFWP-SYKSFDLNL 530
PiCUL1G 460 KLKQQCGGQFTSKMEGMVTDLTLAKENQNHFQEYLSNNPAASPG--IDLTVTVLTTGFWP-SYKSSDLRL 526
PiCUL1C 462 KLKQQCGGQFTSKMEGMVTDLTLARENQASFEEYLSNNPAANPG--IDLTVTVLTTGFWP-SYKSFDLQP 528
PhCUL1 460 KLKQQCGGQFTSKMEGMVTDLTLAKENQNHFQEYLSNNPAASPG--IDLTVTVLTTGFWP-SYKSSDLRL 526
SpCUL1 460 KLKQQCGGQFTSKMEGMVTDLSLVKDNQTHFQEYISNNPATNPG--IDMTVTVLTTGFWP-SYKSCDLNL 526
AtCUL1 458 KLKQQCGGQFTSKMEGMVTDLTLARENQNSFEDYLGSNPAANPG--IDLTVTVLTTGFWP-SYKSFDINL 524
AtCUL2 462 KFKELLGAQFTSKMEGMLTDMTLAKEHQTNFVEFLSVNKTKKLG--MDFTVTVLTTGFWP-SYKTTDLNL 528
AtCUL3A 448 KLKTECGYQFTSKLEGMFTDMKTSEDTMRGFYGSHP---ELSEG--PTLIVQVLTTGSWP-TQPAVPCNL 511
AtCUL3B 448 KLKTECGYQFTSKLEGMFTDMKTSHDTLLGFYNSHP---ELSEG--PTLVVQVLTTGSWP-TQPTIQCNL 511
AtCUL4 510 KLKTECGSQFTNKLEGMFKDIELSKEINESFKQSSQARTKLPSG--IEMSVHVLTTGYWP-TYPPMDVKL 576
OsCUL1-like 464 KLKQQCGGQFTSKMEGMVTDLTVARDHQAKFEEFISTHSELNPG--IALAVTVLTTGFWP-SYKSFDINL 530
OsCUL3-like 451 KLKTECGYQFTSKLEGMFTDMKTSQDTMIDFYAKKSE--ELGDG--PTLDVHILTTGSWP-TQPCPPCNL 515
OsCUL4-like 554 KLKTECGSQFTNKLEGMFKDIELSKEINESFKQSSQARTKLPSG--IEMSVHVLTTGYWP-TYPPMDVKL 620
ScCDC53 506 RLQAANSMEYTGKITKMFQDIRLSKILEDDFAVALKNEPDYSKAKYPDLQPFVLAENMWPFSYQEVEFKL 575
 ..........*.*...*..*.... .. * ... . .. .......*....** ... ....
MdCUL1A 525 PEEMVRCVEVFKSFYETKTKHRKLTWIYSLGTCNVTGKFDTR---PIELVVSTYQAALLLLFNSADKLSY 591
MdCUL1B 492 PAEMVKCVEVFKGFYERKTKCRKITWIYSLGTCNIIGKFELK---EIELVVSTYQGALLLLFNSMDRLSY 558
PbCUL1 531 PPEMVKCVELFREFYQTKTKHRKLTWMYSLGTCNIIGKFEPK---TIELIVTTYQASALLLFNTSDRLSY 597
PavCUL1A 525 PEEMVKCVEVFKGFYETKTKHRKLTWIYSLGTCNVNGKFEPK---NIELVVSTYQAALLLLFNTADKLSY 591
PavCUL1B 531 PAEMVKCVEIFREFYQTKTKHRKLTWMYSLGTCNISGKFEPK---TIELIVTTYQASALLLFNTSDRLSY 597
PiCUL1G 527 PMEMVKCVEVFKEFYQTKTKHRKLTWIYSLGTCHINGKFEPK---TIELVLGTYQAAVLLLFNASDRLSY 593
PiCUL1C 529 PTEMVRCVEVFKEFYQTKTKHRKLTWIYSLGTCNINGKFDPK---TIELVVTTYQASALLLFNASDRLSY 595
PhCUL1 527 PMEMVKCVEVFKEFYQTKTKHRKLTWIYSLGTCHINGKFEPK---TIELVLGTYQAAVLLLFNASDRLSY 593
SpCUL1 527 PVEMAKGVESFKEFYQKKTKHRKLTWIFSLGQCNLNGKFEQK---TIELILGTYQAAALLLFNASDKWSY 593
AtCUL1 525 PSEMIKCVEVFKGFYETKTKHRKLTWIYSLGTCHINGKFDQK---AIELIVSTYQAAVLLLFNTTDKLSY 591
AtCUL2 529 PIEMVNCVEAFKAYYGTKTNSRRLSWIYSLGTCQLAGKFDKK---TIEIVVTTYQAAVLLLFNNTERLSY 595
AtCUL3A 512 PAEVSVLCEKFRSYYLGTHTGRRLSWQTNMGTADIKAIFGKGQ--KHELNVSTFQMCVLMLFNNSDRLSY 579
AtCUL3B 512 PAEVSVLCEKFRSYYLGTHTGRRLSWQTNMGTADIKAVFGKGQ--KHELNVSTFQMCVLMLFNNSDRLSY 579
AtCUL4 577 PHELNVYQDIFKEFYLSKYSGRRLMWQNSLGHCVLKADFSKG---KKELAVSLFQAVVLMLFNDAMKLSF 643
OsCUL1-like 531 PAEMVKCVEVFKEFYQTRTKHRKLTWIYSLGTCNINAKFEAK---TIELIVTTYQAALLLLFNGVDRLSY 597
OsCUL3-like 516 PTEILAICDKFRTYYLGTHSGRRLTWQTNMGTADIKATFGKGQ--KHELNVSTYQMCVLMLFNSTDGLTY 583
OsCUL4-like 621 PHELNVYQDIFKEFYLSKYSGRRLMWQNSLGHCVLKAEFPKG---KKELAVSLFQSVVLMLFNDAQKLSF 687
ScCDC53 576 PKELVPSHEKLKESYSQKHNGRILKWLWPLCRGELKADIGKPGRMPFNFTVTLFQMAILLLYNDADVLTL 645
 * *.. ... ....* .....*...*....... . ... . ... . ..*.. *.*.* .....
MdCUL1A 592 SEILTQLNLTHDDLVRLLHSLSCAKYK-ILTKQPITKTISTNDSFEFNKKFTDKMRRIKIP--------- 651
MdCUL1B 559 SEILSKLNLTHEAAIRVLHSLSCAKYK-ILIKEPDTKTISPNDSFEFNYKFTDRMRRIKIP--------- 618
PbCUL1 598 SEIMTQLNLTDDDVVRLLHSLSCAKYK-ILNKEPNTKTISPTDYFEFNAKFTDKMRRIKIP--------- 657
PavCUL1A 592 SEILTQLNLTHDDLVRLLHSLSCAKYK-ILLKEPNTKTISPTDSFEFNSKFTDRMRRIKIP--------- 651
PavCUL1B 598 SEIMTQLNLTDDDVVRLLHSLSCAKYK-ILNKEPNTKTLSPTDYFEFNSKFTDKMRRIKIP--------- 657
PiCUL1G 594 SDIKSQLNLADDDLVRLLQSLSCAKYK-ILTKDPSNRTVSSTDHFEFNSKFTDKMRRIRVP--------- 653
PiCUL1C 596 QEIMAQLNLSDDDVVRLLHSLSCAKYK-ILNKEPSTKTISQTDVFEFNSKFTDKMRRIKIP--------- 655
PhCUL1 594 SDIKSQLNLADDDLVRLLQSLSCAKYK-ILTKDPSNRTVSSTDHFEFNSKFTDKMRRIRVP--------- 653
SpCUL1 594 ADIKTELNLADDDLVRVLASVSCAKYK-ILNKEPSGRTVSSTDHFEFNSQFTDKMRRIRVP--------- 653
AtCUL1 592 TEILAQLNLSHEDLVRLLHSLSCAKYK-ILLKEPNTKTVSQNDAFEFNSKFTDRMRRIKIP--------- 651
AtCUL2 596 TEILEQLNLGHEDLARLLHSLSCLKYK-ILIKEPMSRNISNTDTFEFNSKFTDKMRRIRVP--------- 655
AtCUL3A 580 KEIEQATEIPAADLKRCLQSLACVKGKNVIKKEPMSKDIGEEDLFVVNDKFTSKFYKVKIGT-------- 641
AtCUL3B 580 KEIEQATEIPTPDLKRCLQSMACVKGKNVLRKEPMSKEIAEEDWFVVNDRFASKFYKVKIGT-------- 641
AtCUL4 644 EDIKDSTSIEDKELRRTLQSLACGKVR-VLQKNPKGRDVEDGDEFEFNDEFAAPLYRIKVN--------- 703
OsCUL1-like 598 SEIVTQLNLSDDDVVRLLHSLSCAKYK-ILSKEPNNRSISPNDVFEFNSKFTDKLRRLKIP--------- 657
OsCUL3-like 584 KDIEQDTAIPASDLKRCLQSLACVKGKNVLRKEPMSKDISEDDTFYFNDKFTSKLVKVKIGT-------- 645
OsCUL4-like 688 LDIKESTGIEDKELRRTLQSLACGKVR-VLQKMPKGRDVEDKDEFVFNEEFSAPLYRIKVN--------- 747
ScCDC53 646 ENIQEGTSLTIQHIAAAMVPFIKFKLIQQVPPG-LDALVKPETQFKLSRPYKALKTNINFASGVKNDILQ 714
 .* .... ...... .....*.. .. ... .... . *... ............

## Slide 4
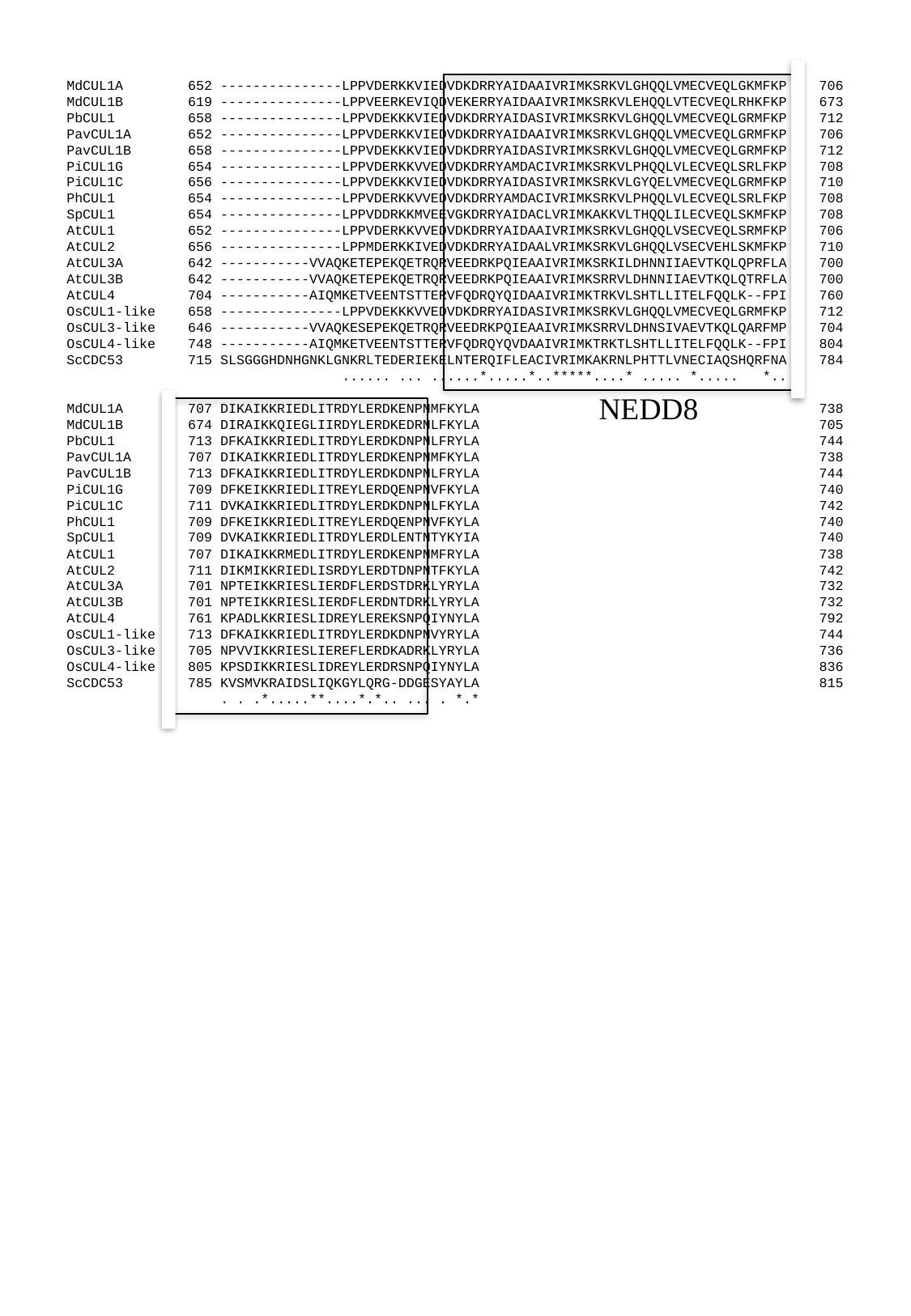

MdCUL1A 652 ---------------LPPVDERKKVIEDVDKDRRYAIDAAIVRIMKSRKVLGHQQLVMECVEQLGKMFKP 706
MdCUL1B 619 ---------------LPPVEERKEVIQDVEKERRYAIDAAIVRIMKSRKVLEHQQLVTECVEQLRHKFKP 673
PbCUL1 658 ---------------LPPVDEKKKVIEDVDKDRRYAIDASIVRIMKSRKVLGHQQLVMECVEQLGRMFKP 712
PavCUL1A 652 ---------------LPPVDERKKVIEDVDKDRRYAIDAAIVRIMKSRKVLGHQQLVMECVEQLGRMFKP 706
PavCUL1B 658 ---------------LPPVDEKKKVIEDVDKDRRYAIDASIVRIMKSRKVLGHQQLVMECVEQLGRMFKP 712
PiCUL1G 654 ---------------LPPVDERKKVVEDVDKDRRYAMDACIVRIMKSRKVLPHQQLVLECVEQLSRLFKP 708
PiCUL1C 656 ---------------LPPVDEKKKVIEDVDKDRRYAIDASIVRIMKSRKVLGYQELVMECVEQLGRMFKP 710
PhCUL1 654 ---------------LPPVDERKKVVEDVDKDRRYAMDACIVRIMKSRKVLPHQQLVLECVEQLSRLFKP 708
SpCUL1 654 ---------------LPPVDDRKKMVEEVGKDRRYAIDACLVRIMKAKKVLTHQQLILECVEQLSKMFKP 708
AtCUL1 652 ---------------LPPVDERKKVVEDVDKDRRYAIDAAIVRIMKSRKVLGHQQLVSECVEQLSRMFKP 706
AtCUL2 656 ---------------LPPMDERKKIVEDVDKDRRYAIDAALVRIMKSRKVLGHQQLVSECVEHLSKMFKP 710
AtCUL3A 642 -----------VVAQKETEPEKQETRQRVEEDRKPQIEAAIVRIMKSRKILDHNNIIAEVTKQLQPRFLA 700
AtCUL3B 642 -----------VVAQKETEPEKQETRQRVEEDRKPQIEAAIVRIMKSRRVLDHNNIIAEVTKQLQTRFLA 700
AtCUL4 704 -----------AIQMKETVEENTSTTERVFQDRQYQIDAAIVRIMKTRKVLSHTLLITELFQQLK--FPI 760
OsCUL1-like 658 ---------------LPPVDEKKKVVEDVDKDRRYAIDASIVRIMKSRKVLGHQQLVMECVEQLGRMFKP 712
OsCUL3-like 646 -----------VVAQKESEPEKQETRQRVEEDRKPQIEAAIVRIMKSRRVLDHNSIVAEVTKQLQARFMP 704
OsCUL4-like 748 -----------AIQMKETVEENTSTTERVFQDRQYQVDAAIVRIMKTRKTLSHTLLITELFQQLK--FPI 804
ScCDC53 715 SLSGGGHDNHGNKLGNKRLTEDERIEKELNTERQIFLEACIVRIMKAKRNLPHTTLVNECIAQSHQRFNA 784
 ...... ... ......*.....*..*****....* ..... *..... *..
MdCUL1A 707 DIKAIKKRIEDLITRDYLERDKENPNMFKYLA 738
MdCUL1B 674 DIRAIKKQIEGLIIRDYLERDKEDRNLFKYLA 705
PbCUL1 713 DFKAIKKRIEDLITRDYLERDKDNPNLFRYLA 744
PavCUL1A 707 DIKAIKKRIEDLITRDYLERDKENPNMFKYLA 738
PavCUL1B 713 DFKAIKKRIEDLITRDYLERDKDNPNLFRYLA 744
PiCUL1G 709 DFKEIKKRIEDLITREYLERDQENPNVFKYLA 740
PiCUL1C 711 DVKAIKKRIEDLITRDYLERDKDNPNLFKYLA 742
PhCUL1 709 DFKEIKKRIEDLITREYLERDQENPNVFKYLA 740
SpCUL1 709 DVKAIKKRIEDLITRDYLERDLENTNTYKYIA 740
AtCUL1 707 DIKAIKKRMEDLITRDYLERDKENPNMFRYLA 738
AtCUL2 711 DIKMIKKRIEDLISRDYLERDTDNPNTFKYLA 742
AtCUL3A 701 NPTEIKKRIESLIERDFLERDSTDRKLYRYLA 732
AtCUL3B 701 NPTEIKKRIESLIERDFLERDNTDRKLYRYLA 732
AtCUL4 761 KPADLKKRIESLIDREYLEREKSNPQIYNYLA 792
OsCUL1-like 713 DFKAIKKRIEDLITRDYLERDKDNPNVYRYLA 744
OsCUL3-like 705 NPVVIKKRIESLIEREFLERDKADRKLYRYLA 736
OsCUL4-like 805 KPSDIKKRIESLIDREYLERDRSNPQIYNYLA 836
ScCDC53 785 KVSMVKRAIDSLIQKGYLQRG-DDGESYAYLA 815
 . . .*.....**....*.*.. ... . *.*
NEDD8
