# Supplementary material for: Identification of SFBB-Containing Canonical and Noncanonical SCF Complexes in Pollen of Apple (Malus × domestica)
Source: PLoS One. 2014 May 21;9(5):e97642. doi: 10.1371/journal.pone.0097642 (PMC4029751; doi:10.1371/journal.pone.0097642)
Supplement: Figure S4 — Amino acid sequence alignment of MdCUL1A and MdCUL1B. Amino acid sequences were aligned using Clustal W. Conserved sites are marked with asterisks. (PPTX) [file pone.0097642.s004.pptx]

## Slide 1
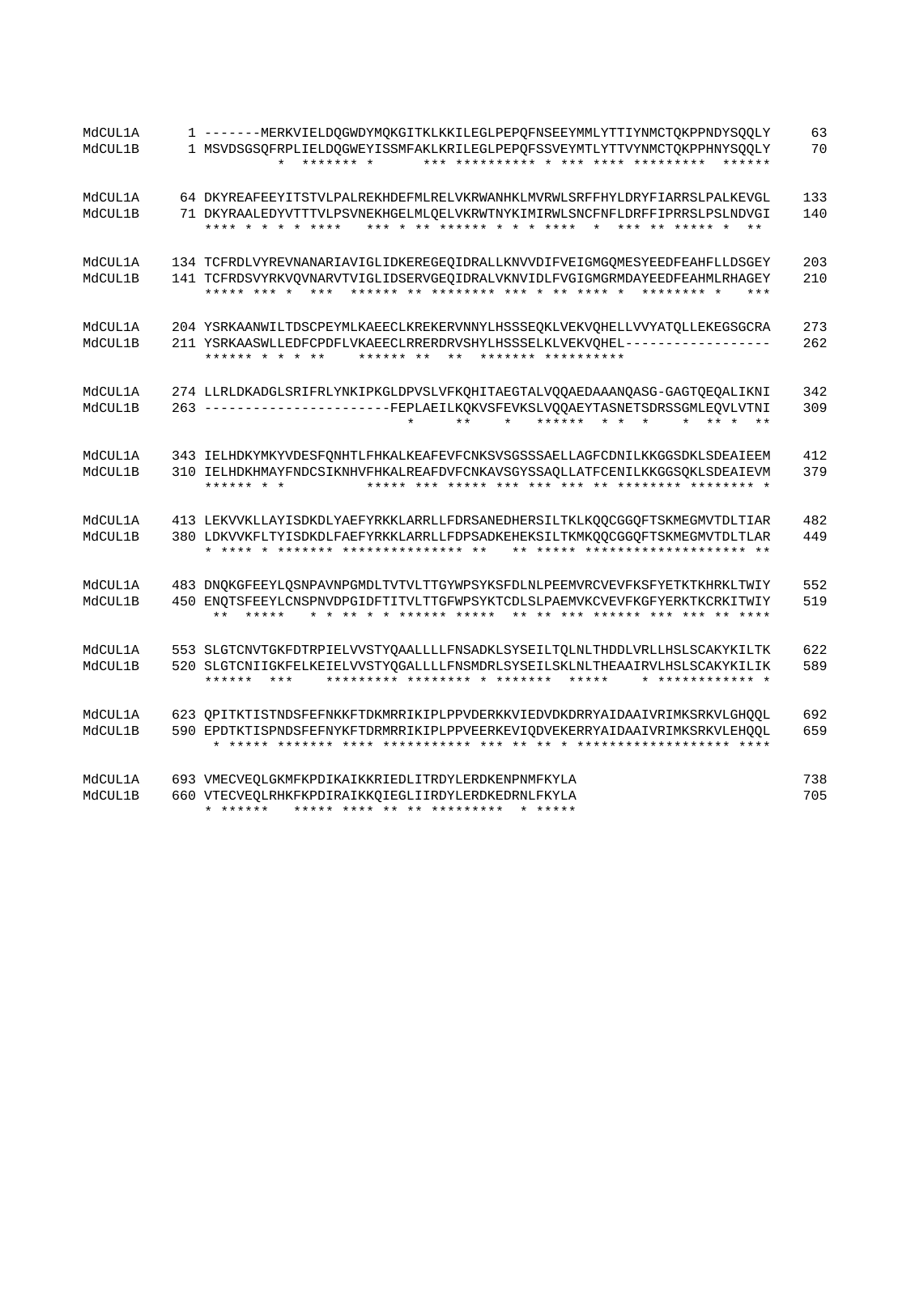

MdCUL1A 1 -------MERKVIELDQGWDYMQKGITKLKKILEGLPEPQFNSEEYMMLYTTIYNMCTQKPPNDYSQQLY 63
MdCUL1B 1 MSVDSGSQFRPLIELDQGWEYISSMFAKLKRILEGLPEPQFSSVEYMTLYTTVYNMCTQKPPHNYSQQLY 70
 * ******* * *** ********** * *** **** ********* ******
MdCUL1A 64 DKYREAFEEYITSTVLPALREKHDEFMLRELVKRWANHKLMVRWLSRFFHYLDRYFIARRSLPALKEVGL 133
MdCUL1B 71 DKYRAALEDYVTTTVLPSVNEKHGELMLQELVKRWTNYKIMIRWLSNCFNFLDRFFIPRRSLPSLNDVGI 140
 **** * * * * **** *** * ** ****** * * * **** * *** ** ***** * **
MdCUL1A 134 TCFRDLVYREVNANARIAVIGLIDKEREGEQIDRALLKNVVDIFVEIGMGQMESYEEDFEAHFLLDSGEY 203
MdCUL1B 141 TCFRDSVYRKVQVNARVTVIGLIDSERVGEQIDRALVKNVIDLFVGIGMGRMDAYEEDFEAHMLRHAGEY 210
 ***** *** * *** ****** ** ******** *** * ** **** * ******** * ***
MdCUL1A 204 YSRKAANWILTDSCPEYMLKAEECLKREKERVNNYLHSSSEQKLVEKVQHELLVVYATQLLEKEGSGCRA 273
MdCUL1B 211 YSRKAASWLLEDFCPDFLVKAEECLRRERDRVSHYLHSSSELKLVEKVQHEL------------------ 262
 ****** * * * ** ****** ** ** ******* **********
MdCUL1A 274 LLRLDKADGLSRIFRLYNKIPKGLDPVSLVFKQHITAEGTALVQQAEDAAANQASG-GAGTQEQALIKNI 342
MdCUL1B 263 -----------------------FEPLAEILKQKVSFEVKSLVQQAEYTASNETSDRSSGMLEQVLVTNI 309
 * ** * ****** * * * * ** * **
MdCUL1A 343 IELHDKYMKYVDESFQNHTLFHKALKEAFEVFCNKSVSGSSSAELLAGFCDNILKKGGSDKLSDEAIEEM 412
MdCUL1B 310 IELHDKHMAYFNDCSIKNHVFHKALREAFDVFCNKAVSGYSSAQLLATFCENILKKGGSQKLSDEAIEVM 379
 ****** * * ***** *** ***** *** *** *** ** ******** ******** *
MdCUL1A 413 LEKVVKLLAYISDKDLYAEFYRKKLARRLLFDRSANEDHERSILTKLKQQCGGQFTSKMEGMVTDLTIAR 482
MdCUL1B 380 LDKVVKFLTYISDKDLFAEFYRKKLARRLLFDPSADKEHEKSILTKMKQQCGGQFTSKMEGMVTDLTLAR 449
 * **** * ******* *************** ** ** ***** ******************** **
MdCUL1A 483 DNQKGFEEYLQSNPAVNPGMDLTVTVLTTGYWPSYKSFDLNLPEEMVRCVEVFKSFYETKTKHRKLTWIY 552
MdCUL1B 450 ENQTSFEEYLCNSPNVDPGIDFTITVLTTGFWPSYKTCDLSLPAEMVKCVEVFKGFYERKTKCRKITWIY 519
 ** ***** * * ** * * ****** ***** ** ** *** ****** *** *** ** ****
MdCUL1A 553 SLGTCNVTGKFDTRPIELVVSTYQAALLLLFNSADKLSYSEILTQLNLTHDDLVRLLHSLSCAKYKILTK 622
MdCUL1B 520 SLGTCNIIGKFELKEIELVVSTYQGALLLLFNSMDRLSYSEILSKLNLTHEAAIRVLHSLSCAKYKILIK 589
 ****** *** ********* ******** * ******* ***** * ************ *
MdCUL1A 623 QPITKTISTNDSFEFNKKFTDKMRRIKIPLPPVDERKKVIEDVDKDRRYAIDAAIVRIMKSRKVLGHQQL 692
MdCUL1B 590 EPDTKTISPNDSFEFNYKFTDRMRRIKIPLPPVEERKEVIQDVEKERRYAIDAAIVRIMKSRKVLEHQQL 659
 * ***** ******* **** *********** *** ** ** * ******************* ****
MdCUL1A 693 VMECVEQLGKMFKPDIKAIKKRIEDLITRDYLERDKENPNMFKYLA 738
MdCUL1B 660 VTECVEQLRHKFKPDIRAIKKQIEGLIIRDYLERDKEDRNLFKYLA 705
 * ****** ***** **** ** ** ********* * *****
