# Supplementary material for: Identification of SFBB-Containing Canonical and Noncanonical SCF Complexes in Pollen of Apple (Malus × domestica)
Source: PLoS One. 2014 May 21;9(5):e97642. doi: 10.1371/journal.pone.0097642 (PMC4029751; doi:10.1371/journal.pone.0097642)
Supplement: Table S1 — Amino acid identities (%) among MdCUL1s and other plant CUL1-like proteins. (PPTX) [file pone.0097642.s005.pptx]

## Slide 1
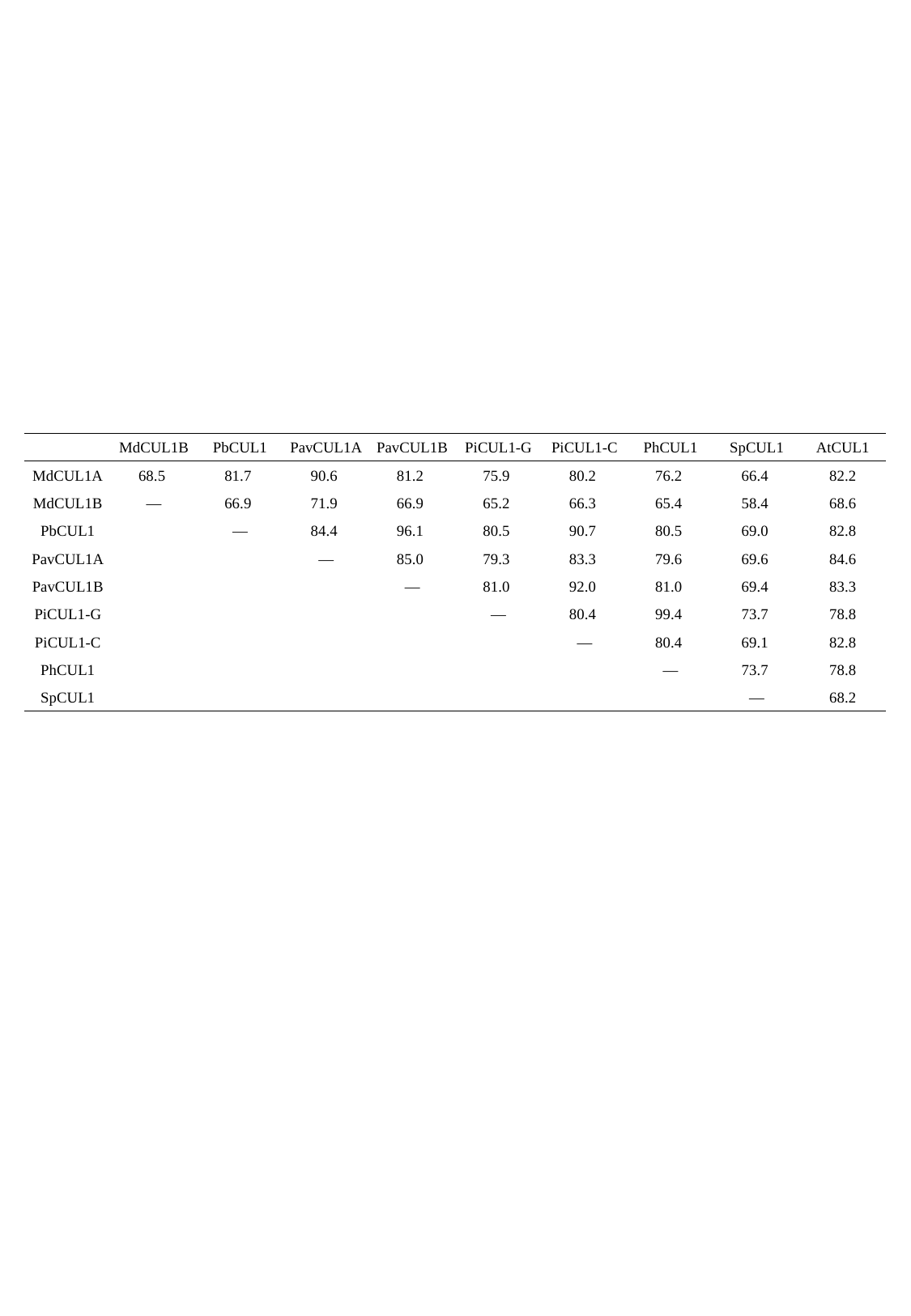

| | MdCUL1B | PbCUL1 | PavCUL1A | PavCUL1B | PiCUL1-G | PiCUL1-C | PhCUL1 | SpCUL1 | AtCUL1 |
| --- | --- | --- | --- | --- | --- | --- | --- | --- | --- |
| MdCUL1A | 68.5 | 81.7 | 90.6 | 81.2 | 75.9 | 80.2 | 76.2 | 66.4 | 82.2 |
| MdCUL1B | ― | 66.9 | 71.9 | 66.9 | 65.2 | 66.3 | 65.4 | 58.4 | 68.6 |
| PbCUL1 | | ― | 84.4 | 96.1 | 80.5 | 90.7 | 80.5 | 69.0 | 82.8 |
| PavCUL1A | | | ― | 85.0 | 79.3 | 83.3 | 79.6 | 69.6 | 84.6 |
| PavCUL1B | | | | ― | 81.0 | 92.0 | 81.0 | 69.4 | 83.3 |
| PiCUL1-G | | | | | ― | 80.4 | 99.4 | 73.7 | 78.8 |
| PiCUL1-C | | | | | | ― | 80.4 | 69.1 | 82.8 |
| PhCUL1 | | | | | | | ― | 73.7 | 78.8 |
| SpCUL1 | | | | | | | | ― | 68.2 |
